# Pre-service teachers’ perceived value of general pedagogical knowledge for practice: Relations with epistemic beliefs and source beliefs

**DOI:** 10.1371/journal.pone.0184971

**Published:** 2017-09-21

**Authors:** Samuel Merk, Tom Rosman, Julia Rueß, Marcus Syring, Jürgen Schneider

**Affiliations:** 1 Institute for Education, University of Tuebingen, Tuebingen, Germany; 2 Leibniz Institute for Psychology Information (ZPID), Trier, Germany; 3 Bologna Lab, Humboldt University, Berlin, Germany; 4 Department of School Education and Rehabilitation, Ludwig-Maximilians-University, Munich, Germany; Waseda University, JAPAN

## Abstract

Pre-service teachers tend to devalue general pedagogical knowledge (GPK) as a valid source for deriving successful teaching practices. The present study investigated beliefs about knowledge sources and epistemic beliefs as predictors for students’ perceived value of GPK. Three pre-registered hypotheses were tested. We expected beliefs that GPK originates from scientific sources to entail a devaluation of GPK (Hypothesis 1). Concerning epistemic beliefs, we expected absolute beliefs to positively, and multiplistic beliefs to negatively predict pre-service teachers’ perceived practical value of GPK (Hypothesis 2). Finally, we expected relationships between epistemic beliefs and pre-service teachers’ perceived practical value of GPK to be confounded by epistemic trustworthiness, perceived topic-specific consistency and topic-specific familiarity (Hypothesis 3). In a study using a split plot design, 365 pre-service teachers were presented with four texts on different educational research topics. For each topic, three text versions were constructed. Even though they were invariant in content, these versions varied in a way that the results were allegedly generated by a practitioner, an expert or by means of a scientific study. Unexpectedly, results showed that research findings allegedly generated by means of a scientific study were associated with a higher perceived value of (topic-specific) GPK for practice (Hypothesis 1). As expected, the perceived value of GPK for practice was predicted by topic-specific multiplism and domain-specific absolutism (Hypothesis 2). These predictive effects were confounded by expertise evaluations of the source and the consistency of prior beliefs with the presented research results (Hypothesis 3). In summary, our results suggest that source beliefs might not be responsible for the devaluation of GPK, but that beliefs on the nature and structure of GPK (i.e., epistemic beliefs) might play an even more important role in this respect. Implications for research and practice are discussed.

## Introduction

General pedagogical knowledge (GPK) is a cornerstone in teacher education policy and practice [1–3]. Nevertheless, many pre-service teachers perceive GPK (e.g., theories of learning) to be irrelevant for classroom practice [4–8]. Research on the factors responsible for this is still in its infancy. This is striking since their perceptions of the value of GPK for practice have been suggested to influence pre-service teachers’ study dropout and motivation to learn [9]. If we understand *why* many pre-service teachers actually devalue GPK, we are able to develop person-specific interventions that address the causes of this devaluation, and adapt curricula and educational practices in a way that fosters students’ motivational engagement. The purpose of this study is to examine whether pre-service teachers’ perceived practical value of GPK may be predicted by their epistemic beliefs as well as by their beliefs about the source of knowledge.

### Perceived practical value of GPK

Teacher education programs worldwide aim at fostering profession-specific knowledge, which is deemed crucial for teaching quality [10–12]. Drawing on Shulman [13,14], current research distinguishes three domains of teacher knowledge [15–18]: content knowledge, pedagogical content knowledge and GPK. The latter incorporates ‘knowledge of theories of learning and general principles of instruction, an understanding of the various philosophies of education, general knowledge about learners, and knowledge of the principles and techniques of classroom management’ ([19], p. 54). GPK is assumed to be highly significant for teaching and learning [1,2]–a claim that has gained empirical support in recent years [3,20,21].

Against this backdrop, teacher educators repeatedly call for enabling and encouraging pre-service teachers to draw on educational theory and research to inform their instructional practices and decision making, as opposed to just acquiring practical tools for teaching or managing classroom situations [22–26]. Thus, pre- and in-service teachers ought to critically read, interpret and understand research knowledge, and use what they find valuable as a basis for their own teaching practices [27–29]. In recent years, this call gained momentum and was reflected in standards for teacher education both at a national level (e.g., for the US, see [30]; for the UK, see [31]; for Australia, see [32]; for Germany, see [33]) and at an international level (for the European Commission, see [34]; for the OECD, see [35]).

Despite such advances, pre-service teachers often perceive research knowledge as being irrelevant for classroom practice [4–8]. This is especially true for GPK: the knowledge conveyed in the respective classes is often regarded as too abstract, too theoretical and too idealistic, and having little to do with the problems that come up in the ‘real’ world of classrooms and schools [36,37]. In short, pre-service teachers tend to view educational theory and research as unrelated to practice and, therefore, as irrelevant for them, which often results in the devaluation and denigration of GPK. At least three major explanations for this are discussed in the current literature:

1. Students’ beliefs of what ought to be learnt: Many pre-service teacher entrants expect to be provided with precise knowledge about what is working best in classroom instruction [20]. For example, they wish to acquire specific teaching tools and strategies that can be directly put into practice without adjustment or modification [38,39]. Correspondingly, Sjølie found pre-service teachers to prefer topics that are highly connected to instructional practices as opposed to the more general topics (e.g., school history) [37]. As she states, pre-service teachers’ primary concern on the direct applicability of GPK represents an ‘instrumentalist view of the relationship between theory and practice’ ([37], p. 740). Such views are often incompatible with how GPK is presented in the field of teacher education, which presumably results in disappointment and the devaluation of GPK [40].
2. Students’ prior beliefs about teaching and learning: Pre-service teachers begin their studies with a set of deep-seated beliefs about teaching and learning that are grounded in years of their own schooling experiences [41,42]. Since such beliefs are fairly rigid and powerful, they likely influence which pedagogical contents the students consider relevant or useful [43,44]. In line with this, several studies demonstrate that pre-service teachers are more likely to accept new pedagogical information if it matches their prior beliefs [37,38,45]. Thus, the devaluation of GPK might partly be due to GPK contradicting students’ initial conceptions of teaching and learning.
3. Students’ beliefs about the source of GPK: Pre-service teachers hold distinct beliefs about different sources of GPK, which basically can be differentiated into practically-derived and theory-based sources of knowledge [9,46]. Practically-derived sources involve personal experiences (i.e., schooling), enactive experiences (i.e., teaching), and observational and vicarious experiences (i.e., observing or learning from practicing teachers). Theory-based sources, by contrast, include formal education (e.g., courses) and formalized bodies of GPK (e.g., educational research literature and textbooks). There is ample evidence that pre-service teachers prefer practically-derived sources over what they are learning during formal coursework at university [38,39,47–49]. For example, they value the testimonies of in-service teachers more than those of researchers or teacher educators, which are often perceived as distant to reality [9,37]. Based on these findings, a new teaching strategy grounded on research may be judged as less valuable than the same strategy supported by in-service teachers [50]. Therefore, the source of knowledge may thus determine what pedagogical information is perceived to be of low (i.e., educational research) or high practical value (i.e., vicarious experiences).

In summary, these lines of discussion indicate that a variety of beliefs held by pre-service teachers are deemed important as they may frame their perceptions on the practical value of GPK and may filter how they experience learning in initial teacher education. This can be supported by recent research on pre-service teachers’ beliefs about the nature of GPK, which are assumed to predict the practical value students ascribe to that knowledge [51]. For example, an individual who views GPK as an accumulation of absolute and certain ‘facts’ might have different perceptions of its practical value than an individual viewing GPK as merely composed of subjective ‘opinions’. In educational psychology, such beliefs have been investigated under the umbrella term *epistemic beliefs* since the 1970s. The following section describes some of the most important frameworks of epistemic beliefs and derives their role for the perceived value of (scientific) GPK.

### Epistemic beliefs

#### Theoretical models of epistemic beliefs

Epistemic beliefs are defined as an individual’s beliefs about the nature of knowledge and knowing [52] and have been shown to significantly affect information processing [53], conceptual change [54], learning [55,56], and academic achievement [57]. Two main approaches can be found in the literature [58]. *Dimensional approaches* call for a fine-grained investigation of different aspects (or dimensions) of epistemic beliefs. A well-known framework by Hofer and Pintrich [52], for example, posits the four distinct dimensions (i) *source of knowledge* (beliefs about where knowledge originates from and how it is derived); (ii) *justification of knowing* (beliefs about how knowledge can be evaluated, personal ideas about evidence and reasoning): (iii) *certainty of knowledge* (beliefs about the stability respectively tentativeness of knowledge): and (iv) *simplicity of knowledge* (beliefs about the complexity or ‘texture’ of knowledge). According to the framework, beliefs that knowledge is complex (simplicity dimension), tentative (certainty dimension), justified by the use of evidence (justification dimension), and originating from the self as knower (source dimension) are more advanced (or ‘sophisticated‘) and thus beneficial for learning [52]. *Developmental approaches*, in contrast, posit a number of broader stages that individuals sequentially pass in their epistemic belief development. According to the framework by Kuhn and Weinstock [59], individuals start out at the *absolutism* stage, in which they conceptualize knowledge in dualistic contrasts, such as right and wrong or truth and untruth [52]. Once this view of knowledge as certain and absolute is dismissed [60], individuals move on to a stage called *multiplism*. Different positions on an issue are now assumed to be equally valid and exchangeable (‘opinions‘). Stressing the subjectivity of knowledge, multiplists are at risk of becoming arbitrary in their views on science (*radical subjectivity*; [52]). The final stage is reached when individuals overcome the paralyzed state of multiplism and realise that ‘viewpoints can be compared and evaluated to assess relative merits‘ ([52], p. 104). In this stage, called *evaluativism*, individuals perceive themselves as being part of the process of knowledge by evaluating and weighting different positions to issues. Even though this is not undisputed [61–63], higher stages are usually deemed as more ‘sophisticated‘ and thus beneficial for learning.

A central advantage of the developmental approach is that its stages can be measured as separate and distinct entities [58,64], which allows a differentiation between multiplistic and evaluativistic stances. In dimensional approaches, by contrast, epistemic ‘sophistication’ is often operationalized as disagreement with absolute statements on the four dimensions presented above. Therefore, using dimensional approaches, researchers cannot differentiate between multiplistic and evaluativistic stances and might ignore an important part of the concept [65]. While this is not as problematic in younger populations (where shifting away from absolutism can be seen as a central developmental task [59]), it poses a significant threat to the validity of corresponding measures in higher education. This is especially true in the social and educational sciences. In fact, compared to absolutism, multiplism is considerably higher in these domains [65,66], and has been shown to impair learning of academic skills (e.g., information literacy) [67].

#### Different levels of specificity of epistemic beliefs

Epistemic beliefs can be conceptualized on different levels. Earlier research [57] almost exclusively used a domain-general approach (i.e., individuals having similar epistemic beliefs across content domains [68]). However, more recent research has challenged this assumption of domain-generality. Since knowledge structures vary considerably between different academic disciplines, Buehl et al. [69] suggest that epistemic beliefs are, to a certain extent, both domain-general *and* discipline-specific. Therefore, they recommend investigating epistemic beliefs on different levels of specificity. In addition, Bråten and Strømsø argue that ‘personal epistemology at different levels of specificity may have the strongest impact on facets of academic learning at comparable levels of specificity’ ([70], p. 640), thus suggesting that the investigation of epistemic beliefs on even more specific levels (i.e., topic-specific epistemic beliefs) also might be worthwhile.

A conceptualization of epistemic beliefs on different levels is also in line with the *Theory of Integrated Domains in Epistemology* (TIDE) by Muis et al. [71], who assume that epistemic beliefs can (and should) be investigated on different, yet interdependent, levels. More specifically, the framework posits that epistemic beliefs are multi-layered (general and discipline-specific and topic-specific), reciprocal depended (more general beliefs influence more specific ones and vice versa), as well as context sensitive, and develop over time [71].

The idea of epistemic beliefs as multi-layered has vast implications for the present research. First, since the concept of practical value of *general pedagogical* knowledge is usually investigated at a discipline-specific level [51], a discipline-specific investigation of epistemic beliefs seems meaningful. Nevertheless, the perceived practical value of knowledge might also vary between different pedagogical topics. This advocates the need to additionally investigate the relationship between epistemic beliefs and the perceived practical value of GPK on a topic-specific level (i.e., by measuring both epistemic beliefs and the perceived practical value of knowledge with regard to a certain topic).

#### Epistemic beliefs and the perceived practical value of GPK

Since both have epistemic components, it seems likely that epistemic beliefs influence pre-service teachers’ perceived practical value of GPK. In this respect, our overarching idea is that both (epistemic beliefs and the practical value of GPK) are embedded into an extensive belief system, and that individuals strive towards consistency in this system [51]. This might be especially true if the respective beliefs are highly contextualized [72]. When closely investigating the theoretical assumptions of the framework given by Kuhn and Weinstock [59], relationships between epistemic beliefs and the perceived practical value of GPK are, therefore, not just likely, but even highly probable.

Students with high absolute beliefs view knowledge as absolute, certain ‘truths’ [59]. Absolutists believe that once a certain pedagogical theory has been verified (an absolutist would probably use the term ‘proven’), its predictions are transferable to all kinds of contexts. This also includes more ‘practical’ contexts, which is why absolute beliefs are consistent with perceptions of a high practical value of GPK. Therefore, we expect absolute beliefs to positively relate to the perceived practical value of GPK. Since absolutists stress expertise as the basis for knowing [52], this might especially apply to knowledge claims originating from an expert source.

In contrast, students with high multiplistic beliefs devalue expertise and view knowledge claims as subjective and arbitrary ‘opinions’ [52]. Knowledge is seen as inconsistent over different knowers, situations and time, which is why a transfer of GPK to practical teaching contexts is not possible, according to a multiplistic perspective [51]. Multiplistic beliefs are thus incompatible with beliefs regarding the practical value of GPK. Therefore, we expect multiplistic beliefs to negatively relate to the perceived practical value of GPK. Since multiplists generally perceive knowledge as inconsistent, we expect this to be true for different sources of information.

Finally, students with high evaluativistic beliefs believe that through evaluating and weighting evidence, it is possible to determine the contexts in which knowledge claims are more (or less) certain. A general expectation that evaluativistic beliefs predict beliefs about the practical value of GPK might be short-sighted. Instead, both the source of a knowledge claim and the topic in question might affect the relationship between evaluativistic beliefs and the perceived practical value of GPK. Evaluativists might believe that claims made by reliable experts and scientific sources have a higher practical value, but only if the topic (or theory) in question allows a transfer of its assumptions to practical contexts.

#### Confounding effects of epistemic trustworthiness, perceived topic-specific consistency and familiarity with the topic

When investigating relationships between epistemic beliefs and the perceived practical value of GPK, controlling for factors that might influence both variables simultaneously is crucial. Perceived familiarity with the knowledge and attitudes are just as relevant in this respect as are individual beliefs about the trustworthiness of experts. In fact, failing to account for such confounding factors is likely to result in so-called spurious correlations, meaning that a third variable explains the relationship between two variables [73].

Epistemic trustworthiness is defined as a set of ‘features of experts that decide whether recipients will depend on and defer to them due to their own limited resources’ ([74], p. 3). It generally includes three dimensions: expertise (e.g., competence or ability), integrity (e.g., honesty and adherence to standards) and benevolence (e.g., good will or intentions) [74]. Since individuals differ in their ascriptions of trustworthiness [75], it is likely that epistemic trustworthiness plays a role in the relationship between epistemic beliefs and the perceived practical value of GPK. For example, individuals holding multiplistic beliefs stress the subjectivity of knowledge and devalue experts [52], thus implicating that they will judge expert claims as less trustworthy. Findings by Strømsø and Bråten [75] support this claim. In their study, students with multiplistic beliefs (i.e., relying on personal interpretations) indeed judged two texts on climate change as less trustworthy than students with more absolute beliefs.

Nevertheless, epistemic trustworthiness is not only prone to be influenced by epistemic beliefs, but also might affect perceptions of the practical value of GPK. In fact, it is hardly plausible that someone who distrusts the author of a text will ascribe high practical value to the knowledge claims included therein. As pointed out above, pre-service teachers tend to ascribe more practical value to the testimony of practitioners rather than on those of researchers or textbook authors [9,37]. Moreover, Gitlin et al. [4] found that pre-service teachers doubted the objectivity of educational research because they perceived findings, in part, to be politically biased. Therefore, we see epistemic trustworthiness as a first confounding variable that should be controlled for when investigating relationships between epistemic beliefs and perceptions of the practical value of GPK.

As stated in the section above, pre-service teachers’ prior beliefs about teaching and learning might influence how they process information and acquire topic-specific knowledge [41]. Several studies have indicated that students’ endorsement of new pedagogical information is stronger if it matches their pre-existing beliefs [37,38,45] since students strive towards consistency in their belief systems [72]. In line with this, if a knowledge claim contradicts an individual’s beliefs on a certain topic (low perceived topic-specific consistency), there is reason to believe that he or she will ascribe less (or no) practical value to these claims. Therefore, we expect a positive relationship between perceived topic-specific consistency and perceived practical value of GPK.

In addition, relationships between topic-specific epistemic beliefs and perceived topic-specific consistency are plausible. Chinn and Brewer [76] describe six ways that individuals discount findings (or data) that contradict their pre-existing beliefs. Among others, individuals are prone to reject such findings by referring to them as random occurrences [76]. This is similar to discounting findings by claiming them to be arbitrary and subjective, which is a central aspect of multiplism [52]. In summary, perceived topic-specific consistency is, therefore, another potential confounding variable worth controlling for.

Perceived and objective prior knowledge on the topics in question has become a somewhat prominent control variable in epistemic belief research. Objective topic knowledge has been shown to relate to text comprehension [77,78] and to judgements of the texts’ trustworthiness [75]. Perceived familiarity of topic has predicted topic-specific certainty [79]. Thus, there is reason to believe that familiarity with the topic also influences the perceived practical value regarding knowledge on this topic. This is in line with findings from social psychology suggesting that threats to one’s self-concept (e.g., due to not knowing much on a topic) leads to self-serving attributions (e.g., to a devaluation of knowledge on this topic) in order to protect one’s self-esteem [80]. Moreover, research suggests that more ‘sophisticated’ epistemic beliefs correlate–although rather inconsistently–with objective topic knowledge [75,81,82]. In sum, controlling for familiarity with the topic also might be meaningful when investigating relationships between epistemic beliefs and the perceived practical value of GPK.

### The current study

The overarching goal of the present article is to analyse both source beliefs and epistemic beliefs as predictors of the perceived practical value of GPK. Therefore, we suggest the following hypotheses, which we pre-registered at the Open Science Framework prior to data collection [83].

Hypothesis 1: The source of knowledge weakly predicts the perceived practical value of GPK. In line with our theorizing above, we expect pre-service teachers to ascribe less practical value to knowledge originating from scientific sources than from experts or practitioners.

Hypothesis 2: Epistemic beliefs are predictors of the perceived practical value of GPK. More specifically, we expect absolute beliefs to positively, and multiplistic beliefs to negatively, correlate with the perceived practical value of GPK; we have no specific expectations regarding evaluativistic beliefs.

Hypothesis 3: These predictive effects of epistemic beliefs are confounded by epistemic trustworthiness, perceived topic-specific consistency and topic knowledge. Therefore, we expect that the predictive effects of epistemic beliefs on perceived practical value of GPK significantly diminish when controlling for epistemic trustworthiness, perceived topic-specific consistency and familiarity with the topic.

## Materials and methods

### Context

The current study was conducted at various universities offering teacher education programs in Germany. In Germany, teacher education is divided into two parts. During the first, university-based part, pre-service teachers study the subjects they will teach later on (usually 2–3 subjects; e.g., history and mathematics) and additionally take several courses on educational and psychological research [40]. During the second part (i.e., after acquiring their Master’s degree), they teach–under the supervision of in-service teachers–at actual schools and reflect their teaching in specialized public institutions for teacher education for an 18 month period. To be admitted for teacher education studies, students require a full maturity secondary certificate (“Abitur”).

### Sample

A study with *N* = 365 pre-service teachers in the first part of their teacher education program (243 males, 119 females, 1 other, 2 missing values; 50.5% in the first two semesters, 31.9% in the third or fourth semester, between 17 and 39 years old, *M* = 21.3, *SD* = 3.1) was conducted. In German teacher education programs, a few students are usually somewhat older since they have already worked in other professions before their studies. With a sample size of over 300, our distribution of age was therefore rather typical.

Even though our participants were in different semesters and had completed varying proportions of their subject-specific studies, all of them were at the same stage of their educational and psychological research curriculum. Moreover, a vast majority of our participants had never engaged in actual teaching. With regard to their subject-specific studies, participants studied a wide range of different subjects, such as Mathematics, History, English, German, Physics, Chemistry, Religion, or Biology. Concerning their educational and psychological research curricula, they had, among others, taken introductory courses in educational science and educational psychology.

### Design

The main part of the questionnaire contained questions pertaining to four short texts, each dealing with a different educational research topic (four-level within-person factor, see Table 1). For every text, three versions were generated, each containing identical information about the topic in question, but simulating different sources of this information (see Table 2). Since the information source was varied between persons (between-person factor ‘source’ with the levels ‘practitioner statement’, ‘expert statement’ and ‘scientific study’), our design resulted in a so called split-plot design [84] (see Table 1). To avoid primacy-recency effects, the sequence of the texts was block randomized.

**Table 1:** Split-plot-design of the study.

	Source of knowledge (between person factor)		
	Practitioner statement	Expert statement	Scientific study
**Educational research topics (within person factor)**	• Text 1: worked out examples • Text 2: cognitive theory of multimedia learning • Text 3: bullying/mobbing • Text 4: classroom size effect on achievement	• Text 1: worked out examples • Text 2: cognitive theory of multimedia learning • Text 3: bullying/mobbing • Text 4: classroom size effect on achievement	• Text 1: worked out examples • Text 2: cognitive theory of multimedia learning • Text 3: bullying/mobbing • Text 4: classroom size effect on achievement

*Note*: The sequence of the four texts was block randomized.

**Table 2:** Excerpts from the intervention text.

Topic: Bullying/mobbing		
Practitioner statement	Expert statement	Scientific study
*During my internship in a middle school*, *I was shocked about how much bullying has spread since my school days*. When I write about bullying, I mean **intentional and repeated negative behaviour of one or more students against another student**. [. . .]*My own experience and the experience of colleagues* show that **about every fourth middle school student and every tenth high school student is being bullied at school**. [. . .]	[. . .] *Experts from the learning sciences define bullying as* **intentional and repeated negative behavior of one or more students against another student**. [. . .] *These Experts ascertain* that **about every fourth middle school student and every tenth high school student is being bullied at school**.	*Our working definition of bullying pertains to Olweus (2010)*, who described it as **intentional and repeated negative behaviour of one or more students against another student**. […] *Fellow researchers have already found out* that **about every fourth middle school student and every tenth high school student is being bullied at school** *(Whitney & Smith*, *1993)*.[. . .]

*Note*: **bold** = invariant text components; *italics* = information pertaining to the source of knowledge.

### Ethics statement

All participants were contacted during lectures, and were informed that participation was voluntary and could be stopped at any time; that the study would take about 40 minutes; and that the data would be stored anonymously. Additionally, at the end of the study, all participants were informed that the text materials were fictitious and did not reflect reality.

As an incentive, each participant was allowed to participate in a lottery of five Amazon vouchers worth €50. The only personal data we assessed were participants’ e-mail addresses in case they agreed to participate in the lottery. E-mail addresses were deleted immediately after the lottery, except for participants who consented to be contacted for a future follow-up study. In this case, e-mail addresses were stored separately from the study data. Moreover, for matching purposes, e-mail addresses were encrypted and stored as salted hashes in the study data.

The study obtained ethics approval by the Ethics Committee of the Economic and Social Science Faculty of the University of Tübingen, and is in full accordance with the Declaration of Helsinki and the APA Ethics Code [85]. The APA Ethics Code explicitly allows to hide the goals of a study if participants will not be exposed to more physical, emotional and financial stress than in their everyday lives. Since this was the case in our study, participants were informed at the end of the study.

### Materials

Multiple steps were taken to design texts that were of high ecological validity and whose content was invariant across information sources. First, we collected curricular valid educational research topics. Subsequently, five researchers who were familiar with the study’s purposes evaluated and selected four topics regarding their representativeness for the domain of educational research and the feasibility of the experimental conditions (i.e., variations in the source of knowledge). Second, we developed invariant text components containing the core information on the topics in question. These invariant components were equal in all three experimental conditions. Third, sentences pertaining to the experimental manipulation (source of knowledge) were added. For the latter two steps, authorship of the textual material was randomly assigned to three of the five researchers. Every text had to satisfy specifications concerning text length (between 130 and 200 words) and text complexity (50 < LIX < 65; [86]). Table 2 shows exemplary excerpts from the texts; all original texts are available as S1 Appendix.

Finally, each text was proofread and edited by one of the five involved researchers who were randomly chosen for this task. In their final versions, text length varied between 139 and 199 words, and text complexity varied between 51.9 and 64.8 LIX.

### Statistical analysis

Due to our within-person factor (text topic), we obtained clustered data for all topic-specific measurements whereby observations on the lower level (= level 1 = topic-level = within-person level) are clustered within persons at the upper level (= level 2 = between-person level). To take into consideration the (potentially) resulting dependence in the data, we used multilevel structural equation modelling (MLSEM) [87]. The central idea of this technique is to decompose a data vector Y_ij_ = Y_T_ (data point of individual j and topic i) into a between-level component Y_B_ = Ȳ_j_ and a within-level component Y_W_ = Y_ij_ − Ȳ_j_. These components are additive and orthogonal (Y_T_ = Y_W_ + Y_B_), which also applies to the population covariance matrices (Σ_T_ = Σ_W_ + Σ_B_). Nevertheless, the estimation of these is more difficult than in the single-level case since the sample between-level covariance matrix is a biased estimator of Σ_B_ [88]. Several approaches have been proposed to solve this issue [89]. In our current investigation, we used a robust variant of the full information maximum likelihood (FIML) estimator implemented in Mplus 7 [90]. This requires modelling the raw data but has the advantage that missing data can be handled model-immanent [91]. Moreover, it corrects standard errors for possible indicator non-normality [92].

The assessment of MLSEM fit indices is still under heavy development [93]. Therefore, we used the established benchmarks from single-level structural equation modelling [94,95] and considered values of the comparative fit index (CFI) and the Tucker-Lewis index (TLI) as indicating acceptable/good fit if they exceeded .90/.95. Values of the root mean square error of approximation (RMSEA) and the standardized root mean error residual (SRMR) were taken to indicate acceptable/good fit if they were under .10/.06 (RMSEA) and .08/.05 (SRMR), respectively [94,95].

As we specified τ–congeneric measurement models within all CFA and MLCFA and since Cronbach’s α assumes more restricted measurement models, we estimated reliability (conceptualized as internal consistency) with McDonald’s ω. Analogously to Cronbach’s α, McDonald’s ω can be interpreted as the proportion of true score variance to total variance (but under the assumption of τ–congeneric measurement models). Hence, values over .70 were considered as acceptable, values over .80 as good, and values over .90 as excellent [96].

In addition to our hypotheses, all data analysis steps were pre-registered in detail prior to data collection at the Open Science Framework [83].

### Between-person measurements

The questionnaire contained between-person level variables that were measured only once as well as within-person level variables that were measured once per topic (= per text), for a total of four measurement points. In the following, we describe all instruments and report the results of (multilevel) confirmatory factor analysis (MCFA/CFA) [97], which allows the estimation of reliability using McDonald’s ω [98] (see section ‘Statistical Analysis’). All items were coded so that high scores correspond to the naming of the scale (e.g., high scores on the topic-specific multiplism scale indicate that an individual has strong multiplistic views on the topics).

#### Epistemic beliefs (EBI-AM)

**Fig 1:**
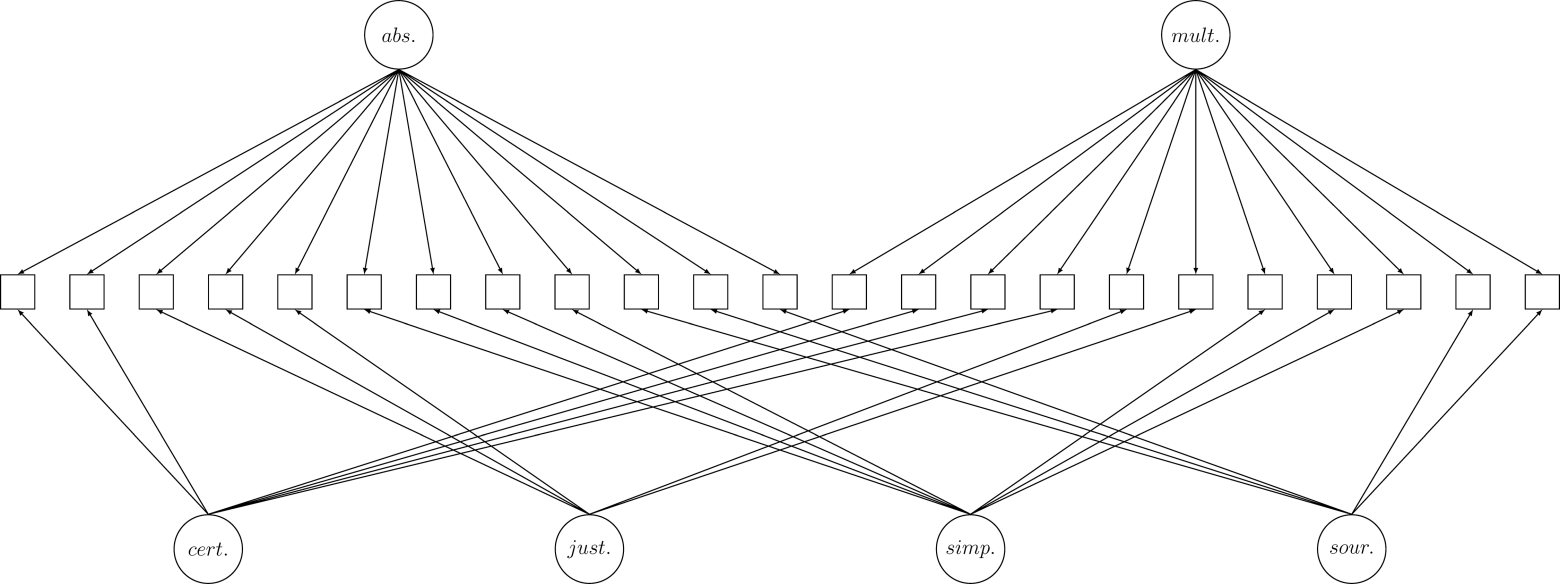
Factor structure of the EBI-AM. Note: Residual and latent covariances are not shown. abs. = absolutism; mult. = multiplism; cert. = certainty; just. = justification; simp. = simplicity; sour. = source.

Absolute and multiplistic epistemic beliefs were measured with an instrument developed by Peter et al. [65], which consisted of 23 items. This instrument allows for measuring epistemological beliefs on a domain-specific level, which is why students were asked to give their answers with the domain of educational science in mind. The items (5-point Likert scale, no inverse formulated items) pertained either to absolute (12 items) or multiplistic beliefs (11 items) and may further be assigned to one of the four dimensions: source, certainty, simplicity and justification (sample item for absolute beliefs regarding certainty: ‘*Truth doesn’t change in this subject*’; sample item for multiplistic beliefs regarding certainty: ‘*In this subject*, *only uncertainty appears to be certain*’). CFA (τ-congeneric measurement models) with two first order factors for the developmental stage (absolute/multiplistic), four first order factors for the dimensions of epistemic beliefs (see Fig 1) and three residual covariances (selected by modifications indices; see the supplemental material for the detailed model specification) resulted in good model fit (χ^2^ = 275.316, *df* = 191, CFI = .930, TLI = .907, RMSEA = .038 (95%CI [0.027, 0.048]), SRMR = .045) and reliability was satisfactory (absolutism: McDonald’s ω = .73, 95% CI [.69, .77]; multiplism: McDonald’s ω = .74, 95% CI [.70, .78]).

#### Epistemic development (FREE)

The level of development of domain-specific epistemic beliefs was further measured using an adapted version of the FREE [64]. The instrument assesses the individual level of development (absolutism, multiplism, evaluativism) based on 13 controversial positions towards well-known educational research topics (example: ‘*It is repeatedly discussed whether grade retention is actually useful or should be abolished’*). For each controversy, participants were presented with three statements (6-point Likert scale, no inverse formulated items) that corresponded to the three levels of development of epistemic beliefs by Kuhn et al. [59]; sample statement for absolutism: ‘*Either grade retention is useful or not! Educational researchers should unequivocally clarify this in the future*’; multiplism: ‘*The expressions for "grade retention" are mere conjecture; no one can really know which factors contribute to school achievement*‘; evaluativism: *‘Even though the experts disagree*, *both may present more or less good reasons for their conceptions*’. The so-called d-index was computed to gain one single score for epistemic development (d = eval − 0.5*mult − 0.5*abs) [64]. CFA with τ-congeneric measurement models (and two freely estimated residual covariances selected by modification indices) justified the choice of such a one-dimensional index (χ^2^ = 98.757, *df* = 63, CFI = .930, TLI = .913, RMSEA = .043, 95%CI_RMSEA_ [0.026, 0.059]), SRMR = .047). The d-index yielded satisfactory reliability (McDonald’s ω = .75, 95% CI [.71, .79])

#### Connotative aspects of epistemic beliefs (CAEB)

Supplementary, connotative aspects of domain-specific epistemic beliefs were assessed with an instrument from Stahl et al. [99]. The instrument was designed as a semantic differential with seven-point bipolar continuums. It contained the dimensions texture (10 items including two inverse formulated items; sample item: ‘*Knowledge in educational science is*. . .*’ ‘structured–unstructured’*, *‘objective–subjective’*) and variability (seven items including five inverse formulated items; sample item: ‘*Knowledge in educational science is*. . .*’ ‘flexible–inflexible’*, *‘complete–open’*). Since the theoretically assumed factor structure could not be confirmed (χ^2^ = 243.305, *df* = 112, CFI = .901, TLI = .880, RMSEA = .060, 95%CI_RMSEA_ [0.049, 0.07], SRMR = .062) with respect to our preregistered inference criteria [83], and the reliability was partly questionable (variability: McDonald’s ω = .63, 95% CI [.56, .68]); texture: McDonald’s ω = .80, 95% CI [.76, .83]), we excluded this measurement from further analysis.

#### Epistemic trustworthiness (METI)

The Muenster Epistemic Trustworthiness Inventory (METI) [74] was used to measure participants’ evaluations of the trustworthiness of the texts’ alleged authors on the dimensions expertise (six items, none inverse formulated), integrity (four items, none inverse formulated), and benevolence (six items, none inverse formulated). This instrument was also designed as a semantic differential (with five steps) and started with ‘*In relation to their insights*, *the authors of the previous texts appear …’* (sample item for expertise: ‘*competent–incompetent’;* integrity: *‘fair–unfair’;* benevolence: ‘*considerate–not considerate’*). The data showed a good fit with regard to a three-dimensional CFA model (τ-congeneric measurement) and three residual covariances (within factors; selected by modification indices; χ^2^ = 192.95, *df* = 70, CFI = .956, TLI = .942, RMSEA = .072, 95%CI_RMSEA_ [0.060, 0.085], SRMR = .054). For all three dimensions, reliability was good (expertise: McDonald’s ω = .88, 95% CI [0.86, 0.90]); integrity: McDonald’s ω = .84, 95% CI [.81, .87]); benevolence: McDonald’s ω = .87, 95% CI [0.84, 0.89]).

#### Treatment check (tc)

We checked if the materials evoked the associations concerning the source of knowledge in the intended way by asking the students for their opinion (6 point Likert scale) about typical activities of the authors of their texts (question prompt: *‘What do you think*: *How frequently do the authors of your texts the following?’*; sample item practitioner: *‘teaching at school’*, sample item expert statement: *‘give advice to schools‘*, sample item scientific study: *‘investigating data’)*. A three-dimensional CFA model (τ-congeneric measurement models) with one freely estimated residual covariance (selected by modification indices; χ^2^ = 103.108, *df* = 23, CFI = .949, TLI = .920, RMSEA = .100, 95%CI_RMSEA_ [0.069, 0.101], SRMR = .068) showed that the absolute model fit was not perfect, but acceptable for the purpose as treatment check. With regard to the length of the scale, reliability was satisfying (practitioner: ω = .85, 95% CI [0.81, .88]); expert statement: ω = .58, 95% CI [.49, .66]); scientific study: ω = .87, 95% CI [.84, .90])). A multiple-indicators multiple-causes (MIMIC) model showed that our treatment worked in the intended way, as source-specific activities could be predicted by the source type with large effect sizes (see S2 Appendix).

### Within-person (topic-specific) measurements

#### Perceived value (pv) of GPK for practice

We assessed students’ topic-specific perceived value of GPK for practice using an adapted version of an established scale from a German large-scale assessment [100]. The scale contains six items (4-point Likert scale including one inverse formulated item) addressing the perceived relevance of the textual information for teacher practice (sample item: ‘*The insights from the text are important for everyday teaching practice’*). Results from a unidimensional MCFA (with τ-congeneric measurement models at both levels) showed a very good model fit (χ^2^ = 58.674, *df* = 18, CFI = .974, TLI = .956, RMSEA = .04, SRMR_within_ = .024, SRMR_between_ = .062) and satisfactory reliability estimates that ranged–depending on the topic in question–between .74 < McDonald’s ω < .78 (union of 95% CI [.70, .78]).

#### Topic-specific multiplism (mult)

Topic-specific epistemic beliefs were measured using the topic-specific multiplism scale [101], which consists of four items (4-point Likert scale, including no inverse formulated items) and pertains to doubts that the findings from the text may be transferrable to other contexts (sample item: *‘The insights from the text are arbitrary*’). A one-dimensional MCFA with τ-congeneric measurement models at both levels and two freely estimated residual covariance parameters (chosen by modification indices) resulted in a very good model fit (χ^2^ = 5.11, *df* = 2, CFI = .996, TLI = .974, RMSEA = .033, SRMR_within_ = .021, SRMR_between_ = .012) and mostly satisfying reliability estimation ranges (.68 < McDonald’s ω < .75; union of 95% CI [.62, .79]).

#### Perceived topic-specific consistency (con)

To measure students’ perceived coherence between text contents and their own views on the topic, we developed a three-item measure (4-point Likert scale, including one inverse formulated item; sample item: ‘*The statements of the just-read text are consistent with my personal opinion on the subject’*). Since an MCFA with independent item clusters resulted in unsatisfactory fit indices for the SRMR at the between level, we fit a model in which the random intercepts were only covaried. This resulted in a very good model fit (χ^2^ = 9.162, *df* = 1, CFI = .994, TLI = .962, RMSEA = .075, SRMR_within_ = .005, SRMR_between_ = .065). The measure further revealed good reliability estimates (.83 < McDonald’s ω < .80; union of 95% CI [.80, .86]).

#### Perceived familiarity with the topic (fam)

We assessed students’ perceived familiarity with the topic in question, referring to [79, 102], with a single item (‘*I am very familiar with the contents of the text I just read*‘; 4-point Likert scale). Please note, that we preregistered this item as “topic-specific knowledge”, but it more strongly deals with *perceived* knowledge respectively *perceived familiarity* with the topic.

## Results

### Predictive effects of source (hypothesis 1)

As mentioned in the methods section, our data were hierarchically clustered because participants were to rate their perceived value of GPK for practice for *four topics*. Therefore, every single data point on the perceived value of GPK for practice could be assigned to a specific topic and these topic-specific responses also could be assigned to a specific person. Therefore, an MCFA model (see the instruments section) was used as the starting point of our investigation of Hypothesis 1.

In model 1 (M1), we extended this MCFA model to a multi-group multilevel confirmatory factor analysis (MGMCFA) to test the measurement invariance of perceived value of GPK for practice over the three experimental groups. In M1, we constrained item loadings at the within-person level as well as item loadings and intercepts at the between-person level. M1 showed good fit to the data (M1: χ^2^ = 166.282, *df* = 87, CFI = .954, TLI = .952, RMSEA = .044, SRMR_within_ = .042, SRMR_between_ = .131), except for the SRMR_between_, which was, even after using modification indices, not satisfying for the between level. Strong or strict measurement invariance between the experimental groups was not established [103]. Therefore, we specified MIMIC models to test our hypotheses.

M2 is such a MIMIC model, which specifies the predictive effects of the source of knowledge (dummy coded with the source ‘practitioner statement’ as a reference category) on the perceived value of GPK for practice. M2 showed a good model fit (χ^2^ = 85.644, *df* = 29, CFI = .966, TLI = .951, RMSEA = .037, SRMR_within_ = .025, SRMR_between_ = .066). The path coefficients of the standardized dummy variables were I^exp^ = 0.085, *p* = .602, 95% CI [−0.184, 0.355] and I^sci^ = 0.635, *p* < .001***, 95% CI [0.381, 0.889], with *R*^2^_between_ = 0.079. This indicates that participants who read texts with the ‘expert statement’ source did not differ from participants in the ‘practitioner statement’ condition with regard to the amount of practical value they ascribed to the texts. Moreover, contradicting the predictions of Hypothesis 1, the group with the ‘scientific study’ source expressed value beliefs of GPK for practice that were 0.635 standard deviations *higher* than those of the reference group. Since this is the opposite of what we expected, Hypothesis 1 is not supported.

### Predictive effects of epistemic beliefs (hypothesis 2)

#### Predictive effects of topic-specific multiplism (within-level) and the FREE (between-level) (M3)

Predictive effects of epistemic beliefs were modelled separately for both levels. At the within-level, we modelled a predictive effect of topic-specific multiplism on topic-specific perceived value of GPK for practice. To make sure that the random intercepts of perceived value of GPK for practice were still interpretable as person-specific means, topic-specific multiplism was centred around its group mean. At the between-person level, we specified a predictive effect of the d-index of the FREE (epistemic development) on the between component (= random intercepts = person specific means) of perceived value of GPK for practice. As shown in Table 3, topic-specific multiplism predicted the within-component of perceived value of GPK for practice significantly and with moderate effect size, whereby the regression coefficients of the d-index were in the expected directions, but missed statistical significance.

**Table 3:** Standardized predictive effects on perceived value of GPK for practice.

	M3	M4	M5	M6	M5a^‡^	M6a^‡^
**Within**						
Topic-specific multiplism	-.432***	-.420***	-.216***	-.206***	-0.222***	-.209***
	(-.489, -.375)	(-.478, -.363)	(-.277, -.155)	(-.267, -.145)	(-.281, -.162)	(-.269, -.149)
Topic-familiarity			.071*	.073*		
			(.007, .135)	(.009, .137)		
Perceived topic-specific consistency			.441***	.449***	0.468***	.474***
			(.381, .502)	(.389, .509)	(.413, 523)	(.419, .528)
*R*^2^_within_	.187	.177	.375	.374	.369	.364
**Between**						
I^exp^	.058	.116	.107	.129	.114	.136
	(-.254, .370)	(-.184, .417)	(-.176, .391)	(-.154, .411)	(-.169, .400)	(-.147, .419)
I^sci^	.599***	.593***	.457**	.434*	.458**	.437**
	(.300, .898)	(.292, .895)	(.166, .747)	(.139, .730)	(.166, .750)	(.141, .734)
d-index (FREE)	-.037		-.066		-.067	
	(-.254, .181)		(-.276, .145)		(-.279, .145)	
Absolutism (EBI-AM)		.467***		.380***		.380**
		(.235, .700)		(.179, .580)		(.179, .580)
Multiplism (EBI-AM)		.138		.153		.160
		(-.068, .344)		(-.027, .333)		(-.021, .340)
Expertise (METI)			-.397***	-.403***	-.389***	-.404***
			(-.543, -.250)	(-.538, -.267)	(-.545, -.252)	(-.540, -.269)
Integrity (METI)			-.027	-.054	-.026	-.053
			(-.172, .117)	(-.195, .087)	(-.171, .118)	(-.195, .088)
Benevolence (METI)			-.126	-.092	-.122	-.090
			(-.274, .021)	(-.235, .051)	(-.270, .025)	(-.234, .053)
d-index * I^exp^	.138		.112		.114	
	(-.055, .331)		(-.066, .291)		(-.065, .293)	
d-index * I^sci^	.150		.116		.115	
	(-.004, .305)		(-.036, .267)		(-.037, .267)	
Absolutism * I^exp^		-.132		-.138		-.140
		(-.324, .059)		(-.301, .023)		(-.301, .021)
Multiplism * I^exp^		.050		.040		.037
		(-.123, .223)		(-.115, .195)		(-.118, .192)
Absolutism * I^sci^		-.234**		-.188*		-.188*
		(-.425, -.042)		(-.357, -.018)		(-.357, -.019)
Multiplism * I^sci^		-.067		-.062		-.065
		(-.233, .100)		(-.205, .082)		(-.209, .079)
*R*^2^_between_	.101	.161	.302	.353	.301	.353

*Note*: *N* = 365; I^exp^ = dummy variable contrasting the ‘expert statement’ with the ‘practitioner statement’ group; I^sci^ = dummy variable contrasting the ‘scientific study’ with the ‘practitioner statement’ group. Within-person variables were standardized around their group means, between-person variables around their grand means.

^‡^These models were not explicitly preregistered.

*p < .05

**p < .01

***p < .001.

#### Predictive effects of topic-specific multiplism (within-level) and the EBI-AM (between-level) (M4)

In model 4 (M4), we exchanged the d-index as a predictor of perceived value of GPK for practice by the absolutism and multiplism dimensions of the epistemic beliefs inventory from Peter et al. [65]. On the within-person level, topic-specific multiplism remained a significant predictor of perceived value of GPK for practice. On the between-person level, calculations revealed a significant and positive association between absolutism and perceived value of GPK for practice for the three experimental groups with significant differences between the ‘practitioner statement’ and the ‘scientific study’ group. In contrast, multiplism showed no significant effects.

### Confounders of predictive effects of epistemic beliefs (hypothesis 3)

Finally, we were interested in potential confounders of the previously found predictive effects of epistemic beliefs on the perceived value of GPK for practice. Therefore, we extended M3 and M4 with predictive effects of perceived topic-specific consistency and familiarity with the topic at the within-level, as well as expertise, integrity and benevolence (the three dimensions of epistemic trustworthiness) at the between level.

#### Confounders of predictive effects of topic-specific multiplism (within-level) and the FREE (d-index; between-level) (M5, M5a)

**Fig 2:**
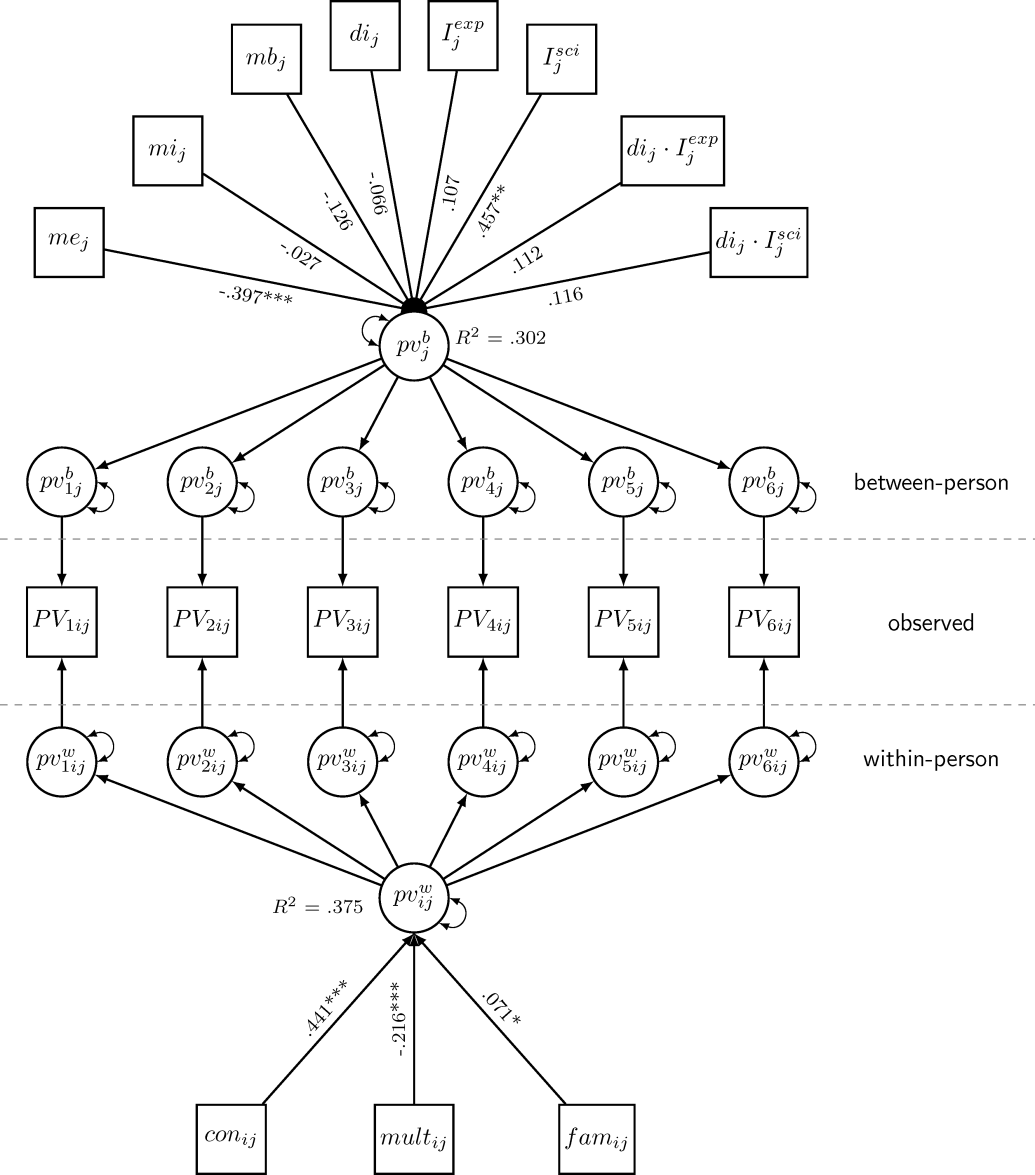
Standardized effects of confounders, topic-specific multiplism and the FREE (M5). *Note*: χ^2^ = 148.954, *df* = 74, CFI = 0.967, TLI = 0.957, RMSEA = 0.027, SRMR_within_ = 0.026, SRMR_between_ = 0.043, con = topic-specific consistency; di = d-index (FREE); I^exp^ = dummy variable contrasting the ‘expert statement’ with the ‘practitioner statement’ group; I^sci^ = dummy variable contrasting the ‘scientific study’ with the ‘practitioner statement’ group; fam = topic familiarity; mb = benevolence (METI); me = expertise (METI); mi = integrity (METI); mult = topic-specific multiplism; pv = perceived value of GPK for practice. *p < .05, **p < .01, ***p < .001.

Adding predictive effects of perceived topic-specific consistency and topic familiarity to M3 (within-person level) resulted in a still significant but much smaller predictive effect of topic-specific multiplism on the perceived value of GPK for practice; a significant effect of moderate size of perceived topic-specific consistency on perceived value of GPK for practice; and a significant but very small effect for topic familiarity (M5; see Fig 2). At the between-person level, the expertise dimension of the epistemic trustworthiness predicted the perceived value of GPK for practice significantly and with moderate effect size. Finally, since assessing topic familiarity with a single item has some methodological shortcomings (e.g., internal consistency cannot be tested), we fit M5 a second time omitting this variable (M5a, χ^2^ = 130.51, *df* = 69, CFI = 0.973, TLI = 0.965, RMSEA = 0.025, SRMR_within_ = 0.024, SRMR_between_ = 0.044). This model (M5a), which was not preregistered, had nearly identical parameters and fit indices (see Table 3).

#### Confounders of predictive effects of topic-specific multiplism (within-level) and the EBI-AM (between-level) (M6)

**Fig 3:**
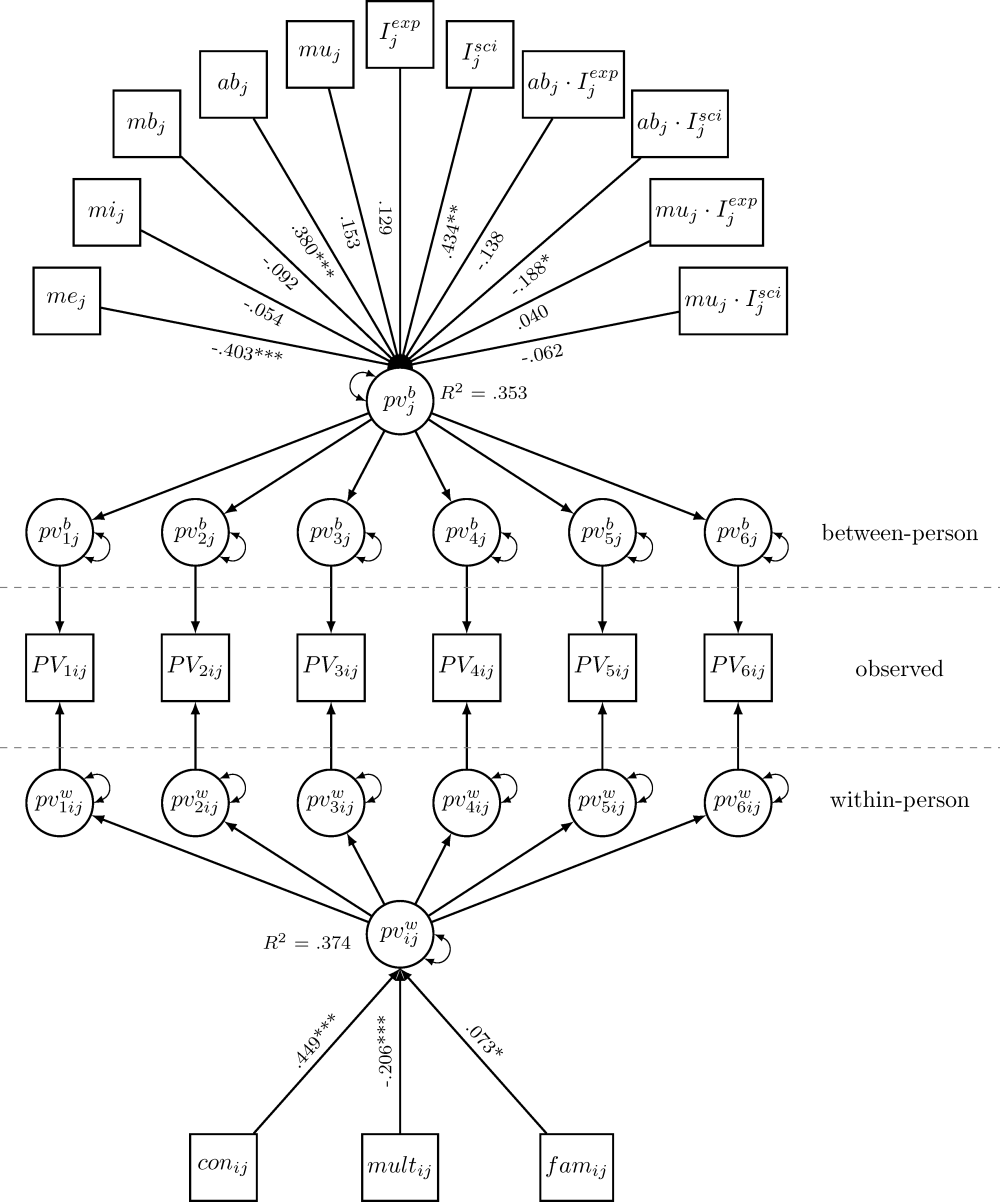
Standardized effects of confounders, topic-specific multiplism and the EBI-AM (M5). *Note*: χ^2^ = 181.307, *df* = 89, CFI = 0.96, TLI = 0.948, RMSEA = 0.027, SRMR_within_ = 0.026, SRMR_between_ = 0.047, ab = absolutism (EBI-AM); con = topic-specific consistency; I^exp^ = dummy variable contrasting the ‘expert statement’ with the ‘practitioner statement’ group; I^sci^ = dummy variable contrasting the ‘scientific study’ with the ‘practitioner statement’ group; fam = topic familiarity; mb = benevolence (METI); me = expertise (METI); mi = integrity (METI); mu = multiplism (EBI-AM); mult = topic-specific multiplism; pv = perceived value of GPK for practice. *p < .05, **p < .01, ***p < .001.

Adding these same potential confounders to M4 resulted in absolutism remaining a significant predictor of perceived value of GPK for practice, whereas its effect size decreased (see Fig 3). The effects of the confounders were comparable to those in M5 (see above). Again, we repeated this analysis without familiarity with the topic for the same reasons outlined above. The resulting model M6a (χ^2^ = 161.163, *df* = 84, CFI = 0.966, TLI = 0.957, RMSEA = 0.026, SRMR_within_ = 0.024, SRMR_between_ = 0.048) showed excellent fit and its coefficients were very similar to those of M6 (see Table 3).

## Discussion

This study had the overarching goal to investigate the role of epistemic beliefs and beliefs about knowledge sources of pre-service teachers for their perceived value of GPK for practice. Therefore, we deduced and preregistered a series of hypotheses as well as a data analysis procedure prior to data collection [83]. Our results partially confirm these hypotheses. Contrary to our expectations, pre-service teachers reported a clearly higher practical value of GPK when it allegedly originated from scientific sources. Hypothesis 1 was thus rejected as we expected lower perceptions of the practical value of GPK that allegedly originated from such sources. However, Hypotheses 2 and 3 were largely confirmed. In fact, topic-specific multiplism and domain-specific absolutism were significant but confounded predictors of the perceived value of GPK; however, this was not the case for domain-specific multiplism and the domain-specific development of epistemic beliefs (d-index). In the following paragraphs, we will discuss potential reasons for these inconsistencies and the limitations of our study. Also, we will emphasize the relevance of our results for teacher education.

### Interpretation of results

To investigate the effects of the source of knowledge on pre-service teachers’ perceived practical value of GPK, we designed several invariant text elements containing findings from educational research. The texts containing these elements were varied in a way that the findings were allegedly generated by a practitioner, an expert, or by means of a scientific study (see Table 2). Our treatment check showed that this manipulation was successful. Contrary to our assumption, scientific studies were perceived to have higher practical value compared to a practitioner or expert statement. Therefore, the assumption that pre-service teachers devalue scientific studies *as such* might not hold up in the light of our empirical evidence. When interpreting this unexpected result, one has to take into account that our hypotheses were derived from studies investigating the practical relevance of scientific GPK on a more general, domain-specific level, and thus did not contrast the practical relevance of scientific and experiential sources at the topic level in the way that we did [9,38,39]. Therefore, ‘content-invariance’ (and thus comparability) between these studies and ours are not given since pre-service teachers may have had diverging topics and knowledge sources in mind when asked about the practical value of educational research versus experiential sources [51,102]. In a replication study, this explanation might easily be investigated by asking pre-service teachers about their perceived practical value of GPK on a domain-specific level before assessing it in a ‘content-invariant’ manner–as we did in the present study. Another interpretation of our findings is that lower perceptions of practical value of GPK in pre-service teachers [4–6] simply do not depend on the source of that knowledge (i.e., educational research), but rather on knowledge content. Independent of its source, pre-service teachers might simply find educational knowledge too abstract or too ‘theoretical’ for it to be of high practical value. With its elaborate experimental design, the present study adds to the literature by suggesting that source beliefs might *not* be responsible for the devaluation and denigration of GPK.

A second inconsistency in our findings concerns Hypothesis 2. We expected predictive effects of epistemic beliefs on the perceived practical value of GPK, which were partially confirmed and partially rejected. In the following, we highlight and interpret two particular aspects. First, with regard to our results on the d-index (FREE inventory), a closer look at the CIs of the respective regression weights (d-index*I^sci^ and d-index*I^exp^) suggests that the insignificance was due to reasons of statistical power. Second, simultaneously modelling epistemic beliefs on a topic level and on a more general domain-specific level (which is, by the way, fully in line with the suggestions of the TIDE framework [71]) gave further insight into their impact on the perceived value of GPK. In fact, multiplism assessed at the topic level was more strongly predictive than domain-specific multiplistic beliefs. Items such as, ‘In this subject, only uncertainty appears to be certain’ can be associated with any educational topic [69,102], which is why they are much more ambiguous. In contrast, perceived practical value of GPK and topic-specific multiplism refer to exactly the same topic. These results suggest that it might indeed be worth investigating epistemic beliefs at different levels with possibly stronger predictions of outcomes on the same level of specificity [70].

Finally, we investigated predictors of the perceived practical value of GPK that involved perceptions about the topic or beliefs about the trustworthiness of sources (Hypothesis 3). Two findings are of particular interest. First, the relationships between topic-specific multiplism and the perceived practical value of GPK seem to be confounded with other predictors, such as topic-specific consistency and the perception of expertise related to the source of knowledge. Therefore, the data support our assumption that relationships between topic-specific multiplism and the perceived practical value of GPK depends on whether the presented knowledge is in line with pre-existing concepts about GPK. Second, the effects of domain-specific absolute beliefs on the perceived practical value of knowledge originating from practitioners and scientific studies diminish slightly when controlling for the confounding variables. Students with absolute views on domain specific knowledge perceive statements from a practitioner as especially valued for practice when they assume them to hold high expertise. For these students, the perceived value of pedagogical knowledge originating from scientific studies is at least partly related to the ascribed expertise of the researchers, or to how much the statement is in line with their own views on the topic.

### Limitations, future directions, and relevance for teacher education

Overall, we see a lot of advantages in approaching pre-service teachers’ perceptions of the practical value of GPK on a topic-specific level. Unfortunately, this approach led to one central limitation: despite investing a lot of effort into selecting and generating representative text materials, we cannot decontextualize our findings from the topics we investigated. In other words, generalizing from four specific topics onto GPK as such, might be problematic.

Another contextual factor that we brought into our study was the nature of our texts. For example, we might have had different results if we had used texts that addressed more controversial topics. Nevertheless, our approach had the strength of avoiding the ambiguity of more general items since it allowed to use topic-specific measures. Moreover, it allowed us to use these items’ variance at the person level as domain-specific (between-person) variance [104], which we did using MLSEM. Furthermore, the possibly of problematic topic selection can be easily investigated through replication studies using other topics. This would be especially interesting when varying the target population, which was another limitation of our study, since we primarily assessed pre-service teachers in their first year of study. In fact, studies have shown that students’ epistemic beliefs considerably change over their first few study semesters [105], and one might argue that similar developments occur regarding the perceived practical value of GPK. Replication studies should also put more focus on Hypothesis 3 by using more/different potential confounders and strengthening the operationalisation of the existing measures–especially the single-item on familiarity with the topic. This item is in fact problematic since its validity has not yet been established and since it is not possible to assess its reliability.

Furthermore, concerning future research, scientists should strive to investigate the psychological processes that are behind our findings on Hypothesis 1. Why do pre-service teachers devaluate GPK [36,37], if not for the fact that such knowledge issues from scientific studies (as was shown in our study)? One possible explanation is that pre-service teachers simply perceive the topics that are dealt with in GPK courses as less valuable for practice, at least when compared to topics in other courses. In the present study design, we only varied the information source but kept constant all four topics. Future research might try to keep the information source constant but vary the topics over different experimental conditions (e.g., GPK vs. pedagogical content knowledge vs. content knowledge [13,15]). Finally, concerning Hypothesis 2, future research should strive to identify mediating factors, especially with regard to our finding that topic-specific multiplism negatively impacts the perceived practical value of GPK. For example, multiplism has shown to entail a more superficial processing of text contents [81,106,107]. This, in turn might lead to students not reflecting on the practical implications of GPK.

With regard to teacher education practice, we judge our unexpected results concerning Hypothesis 1 as ‘good news’ since it indicates that it is not scientificness as such that undermines the perceived practical value of GPK. As stated above, knowledge contents (e.g., students finding certain topics too abstract [37]) might be a more important factor in the devaluation of GPK. Therefore, teacher educators should focus on presenting scientific content in a way that its practical implications become obvious. For example, one might have students apply a certain educational theory to certain typical classroom situations, or discuss this theory together with intervention studies derived from it [101,105].

As far as Hypotheses 2 and 3 are concerned, teacher educators should have a look at the highly significant effects of topic-specific multiplism and topic-specific consistency to deduce implications for teacher education practice. They also might have to be cautious when dealing with absolute epistemic beliefs, since such students tend to ascribe less value to scientific studies. For all three aspects, the literature provides a multitude of intervention approaches. For example, for psychology students, Rosman et al. [66] showed that a guided discussion and integration of controversial empirical evidence might well be suited to reduce both absolute and multiplistic epistemic beliefs. Similarly, Muis et al. [108] used constructivist teaching techniques to foster students’ epistemic beliefs in the context of a social sciences statistics class. Central teaching practices were ‘teacher modelling of critical thinking of content, evaluation of multiple approaches to solving problems, and making connections to prior knowledge’ ([108], p. 213). Finally, topic-specific consistency might be enhanced by ‘helping pre-service teachers discover personal theories that have coloured both what they notice in classrooms and the sense they habitually make of it’ ([45], p. 346). Therefore, in order to confront and elaborate pre-service teachers’ initial beliefs about teaching and learning, teacher education should provide opportunities to help students develop their implicit understandings into explicit, theoretically sound beliefs [109,110]. As several studies demonstrate, this development might be enhanced through carefully constructed learning activities that enable pre-service teachers to examine and reflect on prior beliefs, and share and justify them to peers, connect them to ideas presented in class (e.g., by engaging in problem solving of authentic classroom scenarios) and, finally, re-examine their initial assumptions [111,112].

In summary, our results suggest that source beliefs might not be responsible for the devaluation of GPK. In contrast, beliefs on the nature and structure of GPK (i.e., epistemic beliefs) seem to play a crucial role in this respect. Therefore, since these beliefs influence how pre-service teachers create a value of everything we teach them, future research should dig deeper into the young but growing field of pre-service teachers’ personal epistemologies.

## Supporting information

S1 Appendix Intervention texts. The original (german) intervention texts.
(TXT)

S2 Appendix Documentation of analysis. This file contains the R/MPlus syntax and output.
(HTML)
