# Correction: Pre-service teachers' perceived value of general pedagogical knowledge for practice: Relations with epistemic beliefs and source beliefs

**DOI:** 10.1371/journal.pone.0193632

**Published:** 2018-02-23

**Authors:** Samuel Merk, Tom Rosman, Julia Rueß, Marcus Syring, Jürgen Schneider

In the first sentence of the Sample subsection of the Materials and methods section, the numbers of males and females are incorrectly interchanged. The correct sentence is: A study with *N* = 365 pre-service teachers in the first part of their teacher education program (119 males, 243 females, 1 other, 2 missing values; 50.5% in the first two semesters, 31.9% in the third or fourth semester, between 17 and 39 years old, *M* = 21.3, *SD* = 3.1) was conducted.
