# Supplementary material for: Pre-service teachers’ perceived value of general pedagogical knowledge for practice: Relations with epistemic beliefs and source beliefs
Source: PLoS One. 2017 Sep 21;12(9):e0184971. doi: 10.1371/journal.pone.0184971 (PMC5608309; doi:10.1371/journal.pone.0184971)
Supplement: S2 Appendix — This file contains the R/MPlus syntax and output. (HTML) [file pone.0184971.s002.html]

Documentation of Analyses


# Documentation of Analyses

### *Pre-Service Teachers’ Perceived Value of General Pedagogical Knowledge for Practice: Relations with Epistemic Beliefs and Source Beliefs*

# Setup

The following contains the documentation of all analysis conducted for the manuscript entitled **Pre-Service Teachers’ Perceived Value of General Pedagogical Knowledge for Practice: Relations with Epistemic Beliefs and Source Beliefs** that was submitted to the journal **PLoS One**.  
We primarily used the free and open source software `R`. Multilevel confirmatory factor analysis and multilevel structural equation models were estimated with `MPlus 7.1` using the R package `MplusAutomation` as a R leverage.

# Datawrangling

## Import

```
raw_erf_r1 <- read.table("data/erf_r1/daten.csv", sep = ";", header = T)
raw_erf_r2 <- read.table("data/erf_r2/daten.csv", sep = ";", header = T)
raw_erf_r3 <- read.table("data/erf_r3/daten.csv", sep = ";", header = T)
raw_erf_r4 <- read.table("data/erf_r4/daten.csv", sep = ";", header = T)
raw_exp_r1 <- read.table("data/exp_r1/daten.csv", sep = ";", header = T)
raw_exp_r2 <- read.table("data/exp_r2/daten.csv", sep = ";", header = T)
raw_exp_r3 <- read.table("data/exp_r3/daten.csv", sep = ";", header = T)
raw_exp_r4 <- read.table("data/exp_r4/daten.csv", sep = ";", header = T)
raw_wis_r1 <- read.table("data/wis_r1/daten.csv", sep = ";", header = T)
raw_wis_r2 <- read.table("data/wis_r2/daten.csv", sep = ";", header = T)
raw_wis_r3 <- read.table("data/wis_r3/daten.csv", sep = ";", header = T)
raw_wis_r4 <- read.table("data/wis_r4/daten.csv", sep = ";", header = T)
```

## Joining

```
rawdata <- full_join(raw_erf_r1,
           full_join(raw_erf_r2,
           full_join(raw_erf_r3,
           full_join(raw_erf_r4,
           full_join(raw_exp_r1,
           full_join(raw_exp_r2,
           full_join(raw_exp_r3,
           full_join(raw_exp_r4,
           full_join(raw_wis_r1,
           full_join(raw_wis_r2,
           full_join(raw_wis_r3, raw_wis_r4)))))))))))
```

## Recoding

```
#names(rawdata%>%select(ends_with("_ip")))

rawdata <- rawdata%>%
  mutate(source = as.factor(substr(rawdata$Pseudonym, 1,3)),
         te_01_np = tx_02_np_t1,              
         ## CAEB recoding and renaming: te = texture, vr = variability
         te_02_np = tx_04_np_t1,   
         te_03_np = 8 - tx_05_ip_t1,
         te_04_np = tx_06_np_t1, 
         te_05_np = tx_07_np_t1,     
         te_06_np = tx_08_np_t1,     
         te_07_np = tx_09_np_t1,     
         te_08_np = tx_10_np_t1,         
         te_09_np = 8 - tx_11_ip_t1,
         te_10_np = tx_12_np_t1,  
         
         vr_01_np = va_01_np_t1, 
         vr_02_np = 8 - va_02_ip_t1, 
         vr_03_np = 8 - va_03_ip_t1, 
         vr_04_np = 8 - va_04_ip_t1, 
         vr_05_np = va_05_np_t1, 
         vr_06_np = 8 - tx_13_ip_t1, 
         vr_07_np = 8 - va_06_ip_t1, 
         
         si_03_np = 7 - si_03_ip,                ## Study interest
         si_04_np = 7 - si_04_ip,
         si_06_np = 7 - si_06_ip,
         
         sk_03_np = 5 - sk_03_ip,                ## Self-Concept
         
         
         ## Worked out examples text
         dd_we_ie_05_np = 7 - dd_we_ie_05_ip,    ## Interest-Enjoy
         dd_we_tp_02_np = 5 - dd_we_tp_02_ip,    ## Theory-Practice-Relation
         dd_we_tp_04_np =     dd_we_tp_04_ip,
         dd_we_tp_06_np =     dd_we_tp_06_ip, 
         dd_we_ko_02_np = 5 - dd_we_ko_02_ip,    ## Coherence
         dd_we_ko_04_np = 5 - dd_we_ko_04_ip, 

         ## CTM text
         dd_cm_ie_05_np = 7 - dd_cm_ie_05_ip,    ## Interest-Enjoy
         dd_cm_tp_02_np = 5 - dd_cm_tp_02_ip,    ## Theory-Practice-Relation
         dd_cm_tp_04_np =     dd_cm_tp_04_ip,
         dd_cm_tp_06_np =     dd_cm_tp_06_ip, 
         dd_cm_ko_02_np = 5 - dd_cm_ko_02_ip,    ## Coherence
         dd_cm_ko_04_np = 5 - dd_cm_ko_04_ip, 
         
         ## BFLP text
         ed_bp_ie_05_np = 7 - ed_bp_ie_05_ip,    ## Interest-Enjoy
         ed_bp_tp_02_np = 5 - ed_bp_tp_02_ip,    ## Theory-Practice-Relation
         ed_bp_tp_04_np =     ed_bp_tp_04_ip,
         ed_bp_tp_06_np =     ed_bp_tp_06_ip, 
         ed_bp_ko_02_np = 5 - ed_bp_ko_02_ip,    ## Coherence
         ed_bp_ko_04_np = 5 - ed_bp_ko_04_ip, 
         
         ## CLassroom size text
         ed_cs_ie_05_np = 7 - ed_cs_ie_05_ip,    ## Interest-Enjoy
         ed_cs_tp_02_np = 5 - ed_cs_tp_02_ip,    ## Theory-Practice-Relation
         ed_cs_tp_04_np =     ed_cs_tp_04_ip,
         ed_cs_tp_06_np =     ed_cs_tp_06_ip, 
         ed_cs_ko_02_np = 5 - ed_cs_ko_02_ip,    ## Coherence
         ed_cs_ko_04_np = 5 - ed_cs_ko_04_ip,
         
         
        ## D-Index  FREE
         di_01_np = po_01_np - 0.5*(ab_01_np + re_01_np),
         di_02_np = po_02_np - 0.5*(ab_02_np + re_02_np),
         di_03_np = po_03_np - 0.5*(ab_03_np + re_03_np),
         di_04_np = po_04_np - 0.5*(ab_04_np + re_04_np),
         di_05_np = po_05_np - 0.5*(ab_05_np + re_05_np),
         di_06_np = po_06_np - 0.5*(ab_06_np + re_06_np),
         di_07_np = po_07_np - 0.5*(ab_07_np + re_07_np),
         di_08_np = po_08_np - 0.5*(ab_08_np + re_08_np),
         di_09_np = po_09_np - 0.5*(ab_09_np + re_09_np),
         di_10_np = po_10_np - 0.5*(ab_10_np + re_10_np),
         di_11_np = po_11_np - 0.5*(ab_11_np + re_11_np),
         di_12_np = po_12_np - 0.5*(ab_12_np + re_12_np),
         di_13_np = po_13_np - 0.5*(ab_13_np + re_13_np))


# Drop inverse coded original item
rawdata_np <- tbl_df(rawdata%>%
                       select(-ends_with("_ip"),
                              -starts_with("tx_"),
                              -starts_with("va_")
                              )
                     )


# Create dummy variables (sum constrasts) for source
rawdata_np$I_exp <- ifelse(rawdata_np$source == "exp", 1,
                                ifelse(rawdata_np$source == "erf", -1,0))


rawdata_np$I_wis <- ifelse(rawdata_np$source == "wis", 1,
                                ifelse(rawdata_np$source == "erf", -1,0))
```

## Reshape to Long Form & Group Mean Centering

```
rawdata_long_np <- rawdata_np%>%
  mutate(source   = substr(Pseudonym, 1,3),
         rotation = substr(Pseudonym, 5,6))%>%
  gather(withinitem, value, starts_with("dd_") , starts_with("ed_"))%>%
  mutate(paradigm   = substr(withinitem, 1,2),
         topic      = substr(withinitem, 4,5),
         withinitem2 = as.factor(substr(withinitem, 7,14)))%>%
  select(-withinitem)%>%
  select(-starts_with("dd"))%>%
  select(-starts_with("ed"))%>%
  spread(withinitem2, value)%>%
  group_by(Pseudonym)%>%
  mutate(tr_01_pc = tr_01_np - mean(tr_01_np, na.rm = TRUE),   ## Group Mean Centering
         tr_02_pc = tr_02_np - mean(tr_02_np, na.rm = TRUE),
         tr_03_pc = tr_03_np - mean(tr_03_np, na.rm = TRUE),
         tr_04_pc = tr_04_np - mean(tr_04_np, na.rm = TRUE),
         ke_01_pc = ke_01_np - mean(ke_01_np, na.rm = TRUE),
         cl_01_pc = cl_01_np - mean(cl_01_np, na.rm = TRUE),
         cl_02_pc = cl_02_np - mean(cl_02_np, na.rm = TRUE),
         cl_03_pc = cl_03_np - mean(cl_03_np, na.rm = TRUE),
         ie_01_pc = ie_01_np - mean(ie_01_np, na.rm = TRUE),
         ie_02_pc = ie_02_np - mean(ie_02_np, na.rm = TRUE),
         ie_03_pc = ie_03_np - mean(ie_03_np, na.rm = TRUE),
         ie_04_pc = ie_04_np - mean(ie_04_np, na.rm = TRUE),
         ie_05_pc = ie_05_np - mean(ie_05_np, na.rm = TRUE),
         ko_01_pc = ko_01_np - mean(ko_01_np, na.rm = TRUE),
         ko_02_pc = ko_02_np - mean(ko_02_np, na.rm = TRUE),
         ko_03_pc = ko_03_np - mean(ko_03_np, na.rm = TRUE))%>%
  ungroup()

## Create numeric Person identifier for MPlus
rawdata_long_np$IDnum <- as.numeric(as.factor(rawdata_long_np$Pseudonym))  
## Reorder Factor Levels
rawdata_long_np$topic <- factor(rawdata_long_np$topic, levels=c("we", "cm", "bp", "cs"))  
## Convert Factor Levels to numeric factor for MPlus)
rawdata_long_np$sourcenum <- as.numeric(as.factor(rawdata_long_np$source))
```

# Sample description

## Sample size

```
library(ggplot2)
rawdata$not_na <- rowMeans(is.na(rawdata)) # Variable which proportion NA/not NA 
plot(select(rawdata, -Pseudonym, -Welle, -rawid, -source, -zeit, -kurs)$not_na, 
     ylab = "Percentage of Missing Values per Person")     # Variable

# Total sample size
nrow(rawdata)
```

```
## [1] 365
```

```
# Gender ratio
nrow(rawdata %>% filter(sex == 2))         # female
```

```
## [1] 243
```

```
nrow(rawdata %>% filter(sex == 1))         # male
```

```
## [1] 119
```

```
nrow(rawdata %>% filter(sex == 3))         # other
```

```
## [1] 1
```

```
nrow(rawdata %>% filter(is.na(sex) == T))  # missing
```

```
## [1] 2
```

```
# Age
mean(rawdata$age, na.rm = T)
```

```
## [1] 21.29132
```

```
sd(rawdata$age, na.rm = T)
```

```
## [1] 3.118314
```

```
plotly::ggplotly(qplot(y=rawdata$age, x= 1, geom = "boxplot"))
```

```
## We recommend that you use the dev version of ggplot2 with `ggplotly()`
## Install it with: `devtools::install_github('hadley/ggplot2')`
```

```
## Warning: Removed 8 rows containing non-finite values (stat_boxplot).
```

```
# semester
# Percentage of semester 1 or 2
100/sum(!is.na(rawdata$sem))*sum(rawdata$sem <= 2, na.rm = T)
```

```
## [1] 50.54945
```

```
# Percentage of semester 3 or 4
100/sum(!is.na(rawdata$sem))*sum(rawdata$sem <= 4 & rawdata$sem > 2, na.rm = T)
```

```
## [1] 31.86813
```

# Instruments

## Print Functions

```
## Define a print function for MPlus-Output
fpf_mp <- function(x){  
  
  fm_tmp <- readModels(as.character(x))
  
  return(sprintf(
    "χ² = %s, _df_ = %s,
    CFI = %s, TLI = %s, 
    RMSEA = %s, SRMR~within~ = %s,
    SRMR~between~ = %s",
    round(fm_tmp$summaries$ChiSqM_Value,3), 
    fm_tmp$summaries$ChiSqM_DF,
    round(fm_tmp$summaries$CFI,3),
    round(fm_tmp$summaries$TLI,3),
    round(fm_tmp$summaries$RMSEA_Estimate,3),
    round(fm_tmp$summaries$SRMR.Within,3),
    round(fm_tmp$summaries$SRMR.Between,3)
  )
  )
}

## Define a print function for reliability with within person variables
library(dplyr)
rpf_wv <- function(x){  
  
   reldat <- rawdata_long_np%>%
                    select(starts_with(as.character(x)), topic)%>%
                    select(-ends_with("pc"))%>%
                    group_by(topic)%>%
                    do(data.frame(alpha = MBESS::ci.reliability(data.frame(select(.,
                                          starts_with(as.character(x)))))))
                      
  
  return(sprintf(
          "%s < ω < %s (union of 95%% CI [%s, %s])",
          round(min(reldat$alpha.est), 2),
          round(max(reldat$alpha.est), 2),
          round(min(reldat$alpha.ci.lower), 2),
          round(max(reldat$alpha.ci.upper), 2)
          )
  )
}

## Define a print function for lavaan-Output ##############################
fpf_la <- function(x){  

  fm_tmp <- fitmeasures(x)
                    
  return(sprintf(
          "χ² = %s, _df_ = %s, CFI = %s, TLI = %s, RMSEA = %s (95%%CI [%s, %s]), SRMR = %s",
           round(fm_tmp[c("chisq")],3), 
                 fm_tmp[c("df")],
           round(fm_tmp[c("cfi")],3),
           round(fm_tmp[c("tli")],3),
           round(fm_tmp[c("rmsea")],3),
           round(fm_tmp[c("rmsea.ci.lower")],3),
           round(fm_tmp[c("rmsea.ci.upper")],3),
           round(fm_tmp[c("srmr")],3)
             )
       )
}


## Define a reliability print function for between person variables

rpf_bv <- function(x){  
  
   reldat <- rawdata_np%>%
                    select(starts_with(as.character(x)))%>%
                    select(-ends_with("pc"))
   relinfo <- MBESS::ci.reliability(data.frame(select(reldat, 
                                                      starts_with(as.character(x)))))
                      
  
  return(sprintf(
            "ω = %s, 95%% CI [%s, %s])",
            round(relinfo$est, 2),
            round(relinfo$ci.lower, 2),
            round(relinfo$ci.upper, 2)
            )
         )
}
```

## Perceived Value of GPK for Pratice

### MCFA

```
rawdata_long_np$IDnum <- as.numeric(as.factor(rawdata_long_np$Pseudonym))

MCFA_tp_unres <- mplusObject(
  
   TITLE =  "MCFA__tp_unres",
   
   ANALYSIS =  "TYPE = TWOLEVEL;",
    
   VARIABLE =  "USEVARIABLES = tp_01_np tp_02_np tp_03_np tp_04_np 
                tp_05_np tp_06_np;
                CLUSTER = IDnum;",
  
   MODEL =      "%WITHIN%
                 TPW BY tp_01_np tp_02_np tp_03_np tp_04_np tp_05_np tp_06_np;
           
                 %BETWEEN%
                 TPB BY tp_01_np tp_02_np tp_03_np tp_04_np tp_05_np tp_06_np;",
   
   OUTPUT = "Standardized;",
   
   rdata = rawdata_long_np)

MCFA_tp_unres_fit <- mplusModeler(MCFA_tp_unres, "MCFA_tp_unres.dat", run = 1)
```

### Fit Indices of the MCFA:

χ² = 58.674, *df* = 18, CFI = 0.974, TLI = 0.956, RMSEA = 0.04, SRMRwithin = 0.024, SRMRbetween = 0.062

### Reliability Estimates:

0.74 < ω < 0.78 (union of 95% CI [0.7, 0.81])

## Theory-Specific Relativism

```
library(MplusAutomation)
MCFA_tr_unres <- mplusObject(
  
   TITLE =  "MCFA_tr_unres",
   
   ANALYSIS =  "TYPE = TWOLEVEL;",
    
   VARIABLE =  "USEVARIABLES = tr_01_np tr_02_np tr_03_np tr_04_np;
                     CLUSTER = IDnum;",
        
        
   MODEL    =      "%WITHIN%
                    trW BY tr_01_np tr_02_np tr_03_np tr_04_np;
        
                    %BETWEEN%
                    trB BY tr_01_np tr_02_np tr_03_np tr_04_np;
                           TR_03_NP WITH TR_01_NP;
                           TR_04_NP WITH TR_02_NP; ",  
   
   OUTPUT = "Standardized; MODINDICES",
   
   rdata = rawdata_long_np)

MCFA_tr_unres_fit <- mplusModeler(MCFA_tr_unres, "MCFA_tr_unres.dat", run = 1)
```

### Fit Indices of the MCFA:

χ² = 5.11, *df* = 2, CFI = 0.996, TLI = 0.974, RMSEA = 0.033, SRMRwithin = 0.021, SRMRbetween = 0.012

### Reliability Estimates:

0.68 < ω < 0.75 (union of 95% CI [0.62, 0.79])

## Perceived Consistency with the Topic

```
library(MplusAutomation)
MCFA_ko_within <- mplusObject(
  
   TITLE =  "MCFA_ko_within",
   
   ANALYSIS =  "TYPE = TWOLEVEL;",
    
   VARIABLE =  "USEVARIABLES = ko_01_np ko_02_np ko_03_np;
               
                     CLUSTER = IDnum;",
        
   MODEL    =      "%WITHIN%
                    koW BY ko_01_np(1)
                           ko_02_np(1)
                           ko_03_np(1);

                    %Between%
                    ko_01_np WITH ko_02_np ko_03_np;
                    ko_02_np WITH ko_03_np; ",   
   
   OUTPUT = "Standardized; Modindices",
   rdata = rawdata_long_np)

MCFA_ko_within_fit <- mplusModeler(MCFA_ko_within, "MCFA_ko_within.dat", run = 1)
```

### Fit Indices of the MCFA:

χ² = 9.162, *df* = 1, CFI = 994, TLI = 962, RMSEA = .075, SRMRwithin = .005, SRMRbetween = .065

### Reliability Estimates:

```
   reldat_ko <- rawdata_long_np%>%
                    select(starts_with(as.character("ko_")), topic)%>%
                    select(-ends_with("pc"), -ko_04_np)%>%
                    group_by(topic)%>%
                    do(data.frame(alpha = MBESS::ci.reliability(data.frame(
                      select(., starts_with(as.character("ko")))))))
                      
  
sprintf(
          "%s < ω < %s (union of .95%% CI [%s,%s])",
          round(min(reldat_ko$alpha.est), 2),
          round(max(reldat_ko$alpha.est), 2),
          round(min(reldat_ko$alpha.ci.lower), 2),
          round(max(reldat_ko$alpha.ci.upper), 2)
    
  )
```

[1] “0.83 < ω < 0.89 (union of .95% CI [0.79,0.92])”

## Epistemic Development (FREE)

```
di.cfa.model <- "di =~ di_01_np + di_02_np + di_03_np + di_04_np + di_05_np + di_06_np +
                       di_07_np + di_08_np + di_09_np + di_10_np + di_11_np + di_12_np +
                       di_13_np
                       di_04_np ~~ di_05_np
                       di_03_np ~~ di_06_np"
di.cfa.fitted <- cfa(di.cfa.model, data = rawdata_np)
```

### Fit Indices of the CFA:

χ² = 98.757, *df* = 63, CFI = 0.93, TLI = 0.913, RMSEA = 0.043 (95%CI [0.026, 0.059]), SRMR = 0.047

### Reliability Estimates:

ω = 0.75, 95% CI [0.71, 0.8])

## Epistemic Beliefs Inventory (EBI-AM)

```
ebi.cfa.model <- "abs =~ stab_a_27 + stab_a_41 + rech_a_03 + rech_a_06 + rech_a_10 + 
                         komp_a_07 +  komp_a_20 + komp_a_42 + komp_a_39 + quel_a_08 +
                         quel_a_11 + quel_a_35
                  rel =~ stab_r_04 + stab_r_18 + stab_r_23 + stab_r_21 + rech_r_17 + 
                         rech_r_33 + komp_r_22 + komp_r_30 + komp_r_43 + quel_r_09 + 
                         quel_r_34

                  stab =~ stab_a_27 + stab_a_41 + stab_r_04 + stab_r_18
                  rech =~ rech_a_03 + rech_a_06 + rech_a_10 + rech_r_17 + rech_r_33
                  komp =~ komp_a_07 + komp_a_20 + komp_a_42 + komp_a_39 + komp_r_22 +
                          komp_r_30 + komp_r_43
                  quel =~ quel_a_08 + quel_a_11 + quel_a_35 + quel_r_09 + quel_r_34

                  quel_a_11 ~~ quel_a_35
                  stab_r_04 ~~ stab_r_23
                  rech_r_17 ~~ rech_r_33 " 


ebi.cfa.fitted <- cfa(ebi.cfa.model, data = rawdata)
```

### Fit Indices of the CFA:

χ² = 275.316, *df* = 191, CFI = 0.93, TLI = 0.907, RMSEA = 0.038 (95%CI [0.027, 0.048]), SRMR = 0.045

### Reliability Estimates:

- Absolutism: ω = 0.73, 95% CI [0.68, 0.78])
- Relativism: ω = 0.74, 95% CI [0.7, 0.79])

## Connotative Aspects of Epistemic Beliefs (CAEB)

```
caeb.cfa.model <- " tex =~ te_01_np + te_02_np + te_03_np + te_04_np + te_05_np + 
                           te_06_np + te_07_np + te_08_np + te_09_np + te_10_np
                    var =~ vr_01_np + vr_02_np + vr_03_np + vr_04_np + vr_05_np + 
                           vr_06_np + vr_07_np
                           te_06_np ~~ te_10_np
                           te_05_np ~~ te_08_np
                           vr_02_np ~~ vr_04_np
                           te_03_np ~~ vr_03_np 
                           vr_04_np ~~ vr_07_np 
                           te_06_np ~~ te_07_np "


caeb.cfa.fitted <- cfa(caeb.cfa.model, data = rawdata_np)
```

### Fit Indices of the CFA:

χ² = 243.305, *df* = 112, CFI = 0.901, TLI = 0.88, RMSEA = 0.06 (95%CI [0.049, 0.07]), SRMR = 0.062

### Reliability Estimates:

- Variability: ω = 0.63, 95% CI [0.56, 0.69])
- Texture: ω = 0.8, 95% CI [0.77, 0.83])

## Muenster Epistemic Truthworthiness Inventory (METI)

```
meti.cfa.model <- "me =~ me_01_np + me_02_np + me_03_np + me_04_np + me_05_np + me_06_np
                   mi =~ mi_01_np + mi_02_np + mi_03_np + mi_04_np
                   mb =~ mb_01_np + mb_02_np + mb_03_np + mb_04_np

                   mi_01_np ~~ mi_02_np
                   mb_01_np ~~ mb_02_np
                   mi_03_np ~~ mi_04_np
                   mb_03_np ~~ mb_04_np"

meti.cfa.fitted <- cfa(meti.cfa.model, data = rawdata_np)
```

### Fit Indices of the CFA:

χ² = 192.95, *df* = 70, CFI = 0.956, TLI = 0.942, RMSEA = 0.072 (95%CI [0.06, 0.085]), SRMR = 0.054

### Reliability Estimates:

- Expertise: ω = 0.88, 95% CI [0.85, 0.91])
- Integrity: ω = 0.84, 95% CI [0.78, 0.89])
- Benevolence: ω = 0.87, 95% CI [0.84, 0.89])

## Treatment Check

```
rawdata_np$I_exp_d <- ifelse(rawdata_np$source == "exp", 1, 0) 
rawdata_np$I_wis_d <- ifelse(rawdata_np$source == "wis", 1, 0)


tc.cfa.model <- "exp =~ cr_01_np + cr_02_np + cw_02_np.1
                 wis =~ cw_01_np + cw_02_np + cw_02_np.3
                 erf =~ ce_01_np + ce_02_np + cw_02_np.2
                 cw_01_np ~~ ce_01_np"

tc.cfa.fitted <- cfa(tc.cfa.model, data = rawdata_np)


tc.mimic.model <- "exp =~ cr_01_np + cr_02_np + cw_02_np.1
                   wis =~ cw_01_np + cw_02_np + cw_02_np.3
                   erf =~ ce_01_np + ce_02_np + cw_02_np.2
                   cw_01_np ~~ ce_01_np
                 exp ~ I_exp_d + I_wis_d
                 wis ~ I_exp_d + I_wis_d
                 erf ~ I_exp_d + I_wis_d"

tc.mimic.fitted <- sem(tc.mimic.model, data = rawdata_np)
```

### Fit Indices of the CFA:

χ² = 103.108, *df* = 23, CFI = 0.949, TLI = 0.92, RMSEA = 0.1 (95%CI [0.081, 0.121]), SRMR = 0.068

### Reliability Estimates:

- practicioner activities: ω = 0.85, 95% CI [0.81, 0.88])
- expert activities: ω = 0.58, 95% CI [0.49, 0.66])
- researcher activities: ω = 0.87, 95% CI [0.84, 0.9])

### Fit Indices of the MIMIC model:

χ² = 121.845, *df* = 35, CFI = 0.954, TLI = 0.929, RMSEA = 0.085 (95%CI [0.069, 0.101]), SRMR = 0.058

### Standardized Predicitve Effects of the MIMIC Model:

```
pander::pander(standardizedsolution(tc.mimic.fitted, type = "std.lv")%>%
                 filter(rhs == "I_exp_d" | rhs == "I_wis_d")%>%
                 filter(lhs != "I_exp_d" & lhs != "I_wis_d"))
```

| lhs | op | rhs | est.std | se | z | pvalue |
| --- | --- | --- | --- | --- | --- | --- |
| exp | ~ | I\_exp\_d | 1.64 | 0.1195 | 13.72 | 0 |
| exp | ~ | I\_wis\_d | 1.392 | 0.1281 | 10.87 | 0 |
| wis | ~ | I\_exp\_d | 1.456 | 0.07754 | 18.77 | 0 |
| wis | ~ | I\_wis\_d | 1.667 | 0.0693 | 24.06 | 0 |
| erf | ~ | I\_exp\_d | -1.609 | 0.07302 | -22.04 | 0 |
| erf | ~ | I\_wis\_d | -1.607 | 0.07373 | -21.8 | 0 |

# Results

This sections provides the code and output of the analyses we conducted to investigate our hypotheses. Note that the heading the same than in the corresponding article.

## Predictive Effects of Source (H1)

### M1: Multi-Group MCFA Model

```
### MGMCFA Modell _____________________________________________________________________

MGMCFA_tp_strong <- mplusObject(
  
   TITLE =  "MGMCFA_tp_strong",
   
   ANALYSIS =  "TYPE = TWOLEVEL;",
    
   VARIABLE =  "USEVARIABLES = tp_01_np tp_02_np tp_03_np tp_04_np 
                tp_05_np tp_06_np sourcenum;
                GROUPING IS sourcenum (1 = erf 2 = exp 3 = wis);
                CLUSTER = IDnum;",
   

   MODEL =      "%WITHIN%
                 TPW BY tp_01_np tp_02_np tp_03_np tp_04_np tp_05_np tp_06_np;
   
                 %BETWEEN%
                 TPB BY tp_01_np tp_02_np tp_03_np tp_04_np tp_05_np tp_06_np;
   
                 tp_06_np@0   ;",

   OUTPUT = "Standardized MODINDICES(5);",
   rdata = rawdata_long_np)

MGMCFA_tp_strong_fit <- mplusModeler(MGMCFA_tp_strong, "MGMCFA_tp_strong.dat", run = 1)
```

- The fitindices of M1 were: χ² = 166.282, *df* = 87, CFI = 0.954, TLI = 0.952, RMSEA = 0.044, SRMRwithin = 0.042, SRMRbetween = 0.131

### M2: MIMIC Model with Source Indicator Variables as Predictors

```
# Dummyvariables for MIMIC Model 
rawdata_long_np$I_exp <- ifelse(rawdata_long_np$source == "exp", 1, 0)
rawdata_long_np$I_wis <- ifelse(rawdata_long_np$source == "wis", 1, 0)


### MIMIC_pred_source Modell  ____________________________________

MIMIC_pred_source <- mplusObject(
  
   TITLE =  "MIMIC_pred_source",
   
   ANALYSIS =  "TYPE = TWOLEVEL;",
    
   VARIABLE =  "USEVARIABLES = tp_01_np tp_02_np tp_03_np tp_04_np 
                tp_05_np tp_06_np I_exp I_wis;

                BETWEEN = I_exp I_wis;
                CLUSTER = IDnum;",
   

   MODEL =      "%WITHIN%
                 TPW BY tp_01_np tp_02_np tp_03_np tp_04_np tp_05_np tp_06_np;

                 %BETWEEN%
                 TPB BY tp_01_np tp_02_np tp_03_np tp_04_np tp_05_np tp_06_np;
                 tp_06_np@0;
                 TPB ON I_exp I_wis;",
   
   OUTPUT = "Standardized CINTERVAL MODINDICES(5);",
   
   rdata = rawdata_long_np)

MIMIC_pred_source_fit <- mplusModeler(MIMIC_pred_source, "MIMIC_pred_source.dat", run = 1)
```

- The fitindices of M2 were: χ² = 85.644, *df* = 29, CFI = 0.966, TLI = 0.951, RMSEA = 0.037, SRMRwithin = 0.025, SRMRbetween = 0.066
- The standardized (stdy) predictive effects of M2 were:

```
MIMIC_pred_source_results <- readModels("MIMIC_pred_source.out")
```

Reading model: MIMIC\_pred\_source.out

```
pander::pander(MIMIC_pred_source_results$parameters$stdy.standardized %>% 
                 filter(grepl("ON", paramHeader)))
```

| paramHeader | param | est | se | est\_se | pval | BetweenWithin |
| --- | --- | --- | --- | --- | --- | --- |
| TPB.ON | I\_EXP | 0.085 | 0.164 | 0.522 | 0.602 | Between |
| TPB.ON | I\_WIS | 0.635 | 0.154 | 4.118 | 0 | Between |

```
pander::pander(MIMIC_pred_source_results$parameters$ci.stdy.standardized %>% 
                 filter(grepl("ON", paramHeader)) %>% 
                 dplyr::select(-low.5, -low2.5, -up.5, -up2.5))
```

| paramHeader | param | low5 | est | up5 | BetweenWithin |
| --- | --- | --- | --- | --- | --- |
| TPB.ON | I\_EXP | -0.184 | 0.085 | 0.355 | Between |
| TPB.ON | I\_WIS | 0.381 | 0.635 | 0.889 | Between |

- The \(R^2\) were:

```
pander::pander(MIMIC_pred_source_results$parameters$r2 %>% 
                 filter(grepl("TPB", param) | grepl("TPW", param)))
```

| param | est | se | est\_se | pval | BetweenWithin |
| --- | --- | --- | --- | --- | --- |
| TPB | 0.079 | 0.035 | 2.271 | 0.023 | Between |

## Predictive Effects of Epistemic Beliefs (H2)

### M3: Predictive Effects of d-Index

```
rawdata_long_np <-
rawdata_long_np%>%
 mutate(di_gc = scale(rowMeans(data.frame(di_01_np, di_02_np, di_03_np, di_04_np, di_05_np, 
                                  di_06_np, di_07_np, di_08_np, di_09_np, di_10_np,
                                  di_11_np, di_12_np, di_13_np), na.rm = T), scale = F),
        tr_pc = rowMeans(data.frame(tr_01_pc, tr_02_pc, tr_03_pc, tr_04_pc), na.rm = T),
        ## removing outliers in the way we preregistered
        di_gc = ifelse(scale(di_gc) > 3.29, NA, ifelse(scale(di_gc) < -3.29, NA, di_gc)), 
        tr_pc = ifelse(scale(tr_pc) > 3.29, NA, ifelse(scale(tr_pc) < -3.29, NA, tr_pc)),  
        di_gc_exp = di_gc*I_exp,
        di_gc_wis = di_gc*I_wis)


### MGMCFA Modell  _____________________________________________________________________

MIMIC_pred_di_man <- mplusObject(
  
   TITLE =  "MIMIC_pred_di_man",
   
   ANALYSIS =  "TYPE = TWOLEVEL;",
    
   VARIABLE =  "USEVARIABLES = tp_01_np tp_02_np tp_03_np tp_04_np 
                tp_05_np tp_06_np
                tr_pc
                di_gc di_gc_exp di_gc_wis I_exp I_wis;

                WITHIN = tr_pc;
                BETWEEN = di_gc di_gc_exp di_gc_wis I_exp I_wis;
                CLUSTER = IDnum;",
   

   MODEL =      "%WITHIN%
                 TPW BY tp_01_np tp_02_np tp_03_np tp_04_np tp_05_np tp_06_np;
                 TPW ON tr_pc;

                 %BETWEEN%
                 TPB BY tp_01_np tp_02_np tp_03_np tp_04_np tp_05_np tp_06_np;
                 tp_05_np@0;
                 TPB ON di_gc di_gc_exp di_gc_wis I_exp I_wis;",
   
   OUTPUT = "Standardized CINTERVAL MODINDICES(5);",
   
   rdata = rawdata_long_np)

MIMIC_pred_di_man_fit <- mplusModeler(MIMIC_pred_di_man, "MIMIC_pred_di_man.dat", run = 1)
```

- The fitindices of M3 were: χ² = 112.246, *df* = 49, CFI = 0.968, TLI = 0.956, RMSEA = 0.03, SRMRwithin = 0.024, SRMRbetween = 0.055
- The standardized predictive effects of M3 were:

```
MIMIC_pred_di_man_results <- readModels("MIMIC_pred_di_man.out")
```

Reading model: MIMIC\_pred\_di\_man.out

```
pander::pander(MIMIC_pred_di_man_results$parameters$stdyx.standardized %>% 
                 filter(grepl("ON", paramHeader), !grepl("I_EXP", param),
                        !grepl("I_WIS", param)))
```

| paramHeader | param | est | se | est\_se | pval | BetweenWithin |
| --- | --- | --- | --- | --- | --- | --- |
| TPW.ON | TR\_PC | -0.432 | 0.029 | -14.85 | 0 | Within |
| TPB.ON | DI\_GC | -0.037 | 0.111 | -0.33 | 0.742 | Between |
| TPB.ON | DI\_GC\_EXP | 0.138 | 0.098 | 1.405 | 0.16 | Between |
| TPB.ON | DI\_GC\_WIS | 0.15 | 0.079 | 1.909 | 0.056 | Between |

```
pander::pander(MIMIC_pred_di_man_results$parameters$stdy.standardized %>% 
                 filter(grepl("ON", paramHeader), grepl("I_EXP", param) | 
                          grepl("I_WIS", param)))
```

| paramHeader | param | est | se | est\_se | pval | BetweenWithin |
| --- | --- | --- | --- | --- | --- | --- |
| TPB.ON | I\_EXP | 0.058 | 0.159 | 0.363 | 0.717 | Between |
| TPB.ON | I\_WIS | 0.599 | 0.152 | 3.93 | 0 | Between |

- The standardized confidence intervals of M3 were

```
pander::pander(MIMIC_pred_di_man_results$parameters$ci.stdyx.standardized %>% 
                 filter(grepl("ON", paramHeader), !grepl("I_EXP", param),
                        !grepl("I_WIS", param)) %>% 
                 select(-low.5, -low5, -up.5, -up5))
```

| paramHeader | param | low2.5 | est | up2.5 | BetweenWithin |
| --- | --- | --- | --- | --- | --- |
| TPW.ON | TR\_PC | -0.489 | -0.432 | -0.375 | Within |
| TPB.ON | DI\_GC | -0.254 | -0.037 | 0.181 | Between |
| TPB.ON | DI\_GC\_EXP | -0.055 | 0.138 | 0.331 | Between |
| TPB.ON | DI\_GC\_WIS | -0.004 | 0.15 | 0.305 | Between |

```
pander::pander(MIMIC_pred_di_man_results$parameters$ci.stdy.standardized %>% 
                 filter(grepl("ON", paramHeader), grepl("I_EXP", param) |
                          grepl("I_WIS", param)) %>% 
                 dplyr::select(-low.5, -low5, -up.5, -up5))
```

| paramHeader | param | low2.5 | est | up2.5 | BetweenWithin |
| --- | --- | --- | --- | --- | --- |
| TPB.ON | I\_EXP | -0.254 | 0.058 | 0.37 | Between |
| TPB.ON | I\_WIS | 0.3 | 0.599 | 0.898 | Between |

- The \(R^2\) were:

```
pander::pander(MIMIC_pred_di_man_results$parameters$r2 %>% filter(grepl("TPB", param) |
                                                                  grepl("TPW", param)))
```

| param | est | se | est\_se | pval | BetweenWithin |
| --- | --- | --- | --- | --- | --- |
| TPW | 0.187 | 0.025 | 7.424 | 0 | Within |
| TPB | 0.102 | 0.036 | 2.810 | 0.005 | Between |

### M4: Predictive Effects of EBI

```
rawdata_long_np <-
  rawdata_long_np%>%
    mutate(abs_gc = scale(rowMeans(data.frame(stab_a_27, stab_a_41, rech_a_03, rech_a_06,
                                              rech_a_10, komp_a_07, komp_a_20, komp_a_42,
                                              komp_a_39, quel_a_08, quel_a_11, quel_a_35),
                                   na.rm = T), scale = F),
           rel_gc = scale(rowMeans(data.frame(stab_r_04, stab_r_18, stab_r_23, stab_r_21,
                                              rech_r_17, rech_r_33, komp_r_22, komp_r_30,
                                              komp_r_43, quel_r_09, quel_r_34), 
                                   na.rm = T), scale = F),
           abs_gc = ifelse(scale(abs_gc) > 3.29, NA, ifelse(scale(abs_gc) < -3.29, NA,
                                                            abs_gc)), 
           ## removing outliers in the way we preregistered
           rel_gc = ifelse(scale(rel_gc) > 3.29, NA, ifelse(scale(rel_gc) < -3.29, NA,
                                                            rel_gc)),
           abs_gc_exp = abs_gc*I_exp,
           rel_gc_exp = rel_gc*I_exp,
           abs_gc_wis = abs_gc*I_wis,
           rel_gc_wis = rel_gc*I_wis)

### MIMIC_pred_ebi_man Modell  _______________________________________
MIMIC_pred_ebi_man <- mplusObject(
  
   TITLE =  "MIMIC_pred_ebi_man",
   
   ANALYSIS =  "TYPE = TWOLEVEL;",
    
   VARIABLE =  "USEVARIABLES = tp_01_np tp_02_np tp_03_np tp_04_np 
                tp_05_np tp_06_np
                tr_pc
                abs_gc rel_gc abs_gc_exp rel_gc_exp abs_gc_wis rel_gc_wis 
                I_exp I_wis;

                WITHIN =  tr_pc;
                BETWEEN = abs_gc rel_gc abs_gc_exp rel_gc_exp abs_gc_wis rel_gc_wis
                          I_exp I_wis;
                CLUSTER = IDnum;",
   

   MODEL =      "%WITHIN%
                 TPW BY tp_01_np tp_02_np tp_03_np tp_04_np tp_05_np tp_06_np;
                 TPW ON tr_pc;

                 %BETWEEN%
                 TPB BY tp_01_np tp_02_np tp_03_np tp_04_np tp_05_np tp_06_np;
                 tp_05_np@0;
                 TPB ON abs_gc rel_gc abs_gc_exp rel_gc_exp abs_gc_wis rel_gc_wis 
                        I_exp I_wis;
   
   
   ",
   OUTPUT = "Standardized CINTERVAL MODINDICES(5);",
   rdata = rawdata_long_np)

MIMIC_pred_ebi_man_fit <- mplusModeler(MIMIC_pred_ebi_man, "MIMIC_pred_ebi_man.dat", run = 1)
```

- The fitindices of M4 were: χ² = 148.461, *df* = 64, CFI = 0.957, TLI = 0.944, RMSEA = 0.03, SRMRwithin = 0.024, SRMRbetween = 0.057
- The standardized predictive effects of M4 were:

```
MIMIC_pred_ebi_man_results <- readModels("MIMIC_pred_ebi_man.out")
```

Reading model: MIMIC\_pred\_ebi\_man.out

```
pander::pander(MIMIC_pred_ebi_man_results$parameters$stdyx.standardized %>% 
                 filter(grepl("ON", paramHeader), !grepl("I_EXP", param), 
                        !grepl("I_WIS", param)))
```

| paramHeader | param | est | se | est\_se | pval | BetweenWithin |
| --- | --- | --- | --- | --- | --- | --- |
| TPW.ON | TR\_PC | -0.42 | 0.029 | -14.31 | 0 | Within |
| TPB.ON | ABS\_GC | 0.467 | 0.119 | 3.936 | 0 | Between |
| TPB.ON | REL\_GC | 0.138 | 0.105 | 1.31 | 0.19 | Between |
| TPB.ON | ABS\_GC\_EXP | -0.132 | 0.098 | -1.355 | 0.175 | Between |
| TPB.ON | REL\_GC\_EXP | 0.05 | 0.088 | 0.569 | 0.569 | Between |
| TPB.ON | ABS\_GC\_WIS | -0.234 | 0.098 | -2.392 | 0.017 | Between |
| TPB.ON | REL\_GC\_WIS | -0.067 | 0.085 | -0.782 | 0.434 | Between |

```
pander::pander(MIMIC_pred_ebi_man_results$parameters$stdy.standardized %>% 
                 filter(grepl("ON", paramHeader), grepl("I_EXP", param) | 
                          grepl("I_WIS", param)))
```

| paramHeader | param | est | se | est\_se | pval | BetweenWithin |
| --- | --- | --- | --- | --- | --- | --- |
| TPB.ON | I\_EXP | 0.116 | 0.153 | 0.76 | 0.447 | Between |
| TPB.ON | I\_WIS | 0.593 | 0.154 | 3.863 | 0 | Between |

- The standardized confidence intervals of M4 were

```
pander::pander(MIMIC_pred_ebi_man_results$parameters$ci.stdyx.standardized %>% 
                 filter(grepl("ON", paramHeader), !grepl("I_EXP", param), 
                        !grepl("I_WIS", param)) %>% 
                 select(-low.5, -low5, -up.5, -up5))
```

| paramHeader | param | low2.5 | est | up2.5 | BetweenWithin |
| --- | --- | --- | --- | --- | --- |
| TPW.ON | TR\_PC | -0.478 | -0.42 | -0.363 | Within |
| TPB.ON | ABS\_GC | 0.235 | 0.467 | 0.7 | Between |
| TPB.ON | REL\_GC | -0.068 | 0.138 | 0.344 | Between |
| TPB.ON | ABS\_GC\_EXP | -0.324 | -0.132 | 0.059 | Between |
| TPB.ON | REL\_GC\_EXP | -0.123 | 0.05 | 0.223 | Between |
| TPB.ON | ABS\_GC\_WIS | -0.425 | -0.234 | -0.042 | Between |
| TPB.ON | REL\_GC\_WIS | -0.233 | -0.067 | 0.1 | Between |

```
pander::pander(MIMIC_pred_ebi_man_results$parameters$ci.stdy.standardized %>% 
                 filter(grepl("ON", paramHeader), grepl("I_EXP", param) |
                          grepl("I_WIS", param)) %>% 
                 dplyr::select(-low.5, -low5, -up.5, -up5))
```

| paramHeader | param | low2.5 | est | up2.5 | BetweenWithin |
| --- | --- | --- | --- | --- | --- |
| TPB.ON | I\_EXP | -0.184 | 0.116 | 0.417 | Between |
| TPB.ON | I\_WIS | 0.292 | 0.593 | 0.895 | Between |

- The \(R^2\) were:

```
pander::pander(MIMIC_pred_ebi_man_results$parameters$r2 %>% filter(grepl("TPB", param) |
                                                                     grepl("TPW", param)))
```

| param | est | se | est\_se | pval | BetweenWithin |
| --- | --- | --- | --- | --- | --- |
| TPW | 0.177 | 0.025 | 7.156 | 0 | Within |
| TPB | 0.161 | 0.049 | 3.295 | 0.001 | Between |

## Confounders of Predictive Effects of Epistemic Beliefs

### M5: Confounders of Predictive Effects d-Index (H3)

```
### Compute arithemtic means of scales  ____________________________________
rawdata_long_np <- rawdata_long_np%>%
  mutate(cl_pc = rowMeans(data.frame(cl_01_pc, cl_02_pc, cl_03_pc), na.rm = T),
         ko_pc = rowMeans(data.frame(ko_01_pc, ko_02_pc, ko_03_pc), na.rm = T),
         me_np = rowMeans(data.frame(me_01_np, me_02_np, me_03_np, me_04_np, me_05_np, me_06_np), na.rm = T),
         mi_np = rowMeans(data.frame(mi_01_np, mi_02_np, mi_03_np, mi_04_np), na.rm = T),
         mb_np = rowMeans(data.frame(mb_01_np, mb_02_np, mb_03_np, mb_04_np), na.rm = T),
         ko_pc = ifelse(scale(ko_pc) > 3.29, NA,ifelse(scale(ko_pc) < -3.29, NA, ko_pc)),
         me_np = ifelse(scale(me_np) > 3.29, NA,ifelse(scale(me_np) < -3.29, NA, me_np)),
         mi_np = ifelse(scale(mi_np) > 3.29, NA,ifelse(scale(mi_np) < -3.29, NA, mi_np)),
         mb_np = ifelse(scale(mb_np) > 3.29, NA,ifelse(scale(mb_np) < -3.29, NA, mb_np)))


### MIMIC_conf_di_man Modell  _________________________________
MIMIC_conf_di_man <- mplusObject(
  
   TITLE =  "MIMIC_conf_di_man",
   
   ANALYSIS =  "TYPE = TWOLEVEL;",
    
   VARIABLE =  "USEVARIABLES = tp_01_np tp_02_np tp_03_np tp_04_np 
                tp_05_np tp_06_np
                tr_pc ke_01_pc ko_pc 
                di_gc di_gc_exp di_gc_wis I_exp I_wis me_np mi_np mb_np;

                WITHIN =  tr_pc ke_01_pc ko_pc;
                BETWEEN = di_gc di_gc_exp di_gc_wis I_exp I_wis me_np mi_np mb_np;
                CLUSTER = IDnum;",
   

   MODEL =      "%WITHIN%
                 TPW BY tp_01_np tp_02_np tp_03_np tp_04_np tp_05_np tp_06_np;

                 TPW ON tr_pc ke_01_pc ko_pc ;

                 %BETWEEN%
                 TPB BY tp_01_np tp_02_np tp_03_np tp_04_np tp_05_np tp_06_np;
                 tp_05_np@0;
                 TPB ON di_gc di_gc_exp di_gc_wis I_exp I_wis me_np mi_np mb_np; ",
   
   OUTPUT = "Standardized CINTERVAL MODINDICES(5);",
   
   rdata = rawdata_long_np)

MIMIC_conf_di_man_fit <- mplusModeler(MIMIC_conf_di_man, "MIMIC_conf_di_man.dat", run = 1)
```

- The fitindices of M5 were: χ² = 148.954, *df* = 74, CFI = 0.967, TLI = 0.957, RMSEA = 0.027, SRMRwithin = 0.026, SRMRbetween = 0.043
- The standardized predictive effects of M5 were:

```
MIMIC_conf_di_man_results <- readModels("MIMIC_conf_di_man.out")
```

Reading model: MIMIC\_conf\_di\_man.out

```
pander::pander(MIMIC_conf_di_man_results$parameters$stdyx.standardized %>% 
                 filter(grepl("ON", paramHeader), !grepl("I_EXP", param), 
                        !grepl("I_WIS", param)))
```

| paramHeader | param | est | se | est\_se | pval | BetweenWithin |
| --- | --- | --- | --- | --- | --- | --- |
| TPW.ON | TR\_PC | -0.216 | 0.031 | -6.951 | 0 | Within |
| TPW.ON | KE\_01\_PC | 0.071 | 0.033 | 2.172 | 0.03 | Within |
| TPW.ON | KO\_PC | 0.441 | 0.031 | 14.24 | 0 | Within |
| TPB.ON | DI\_GC | -0.066 | 0.107 | -0.613 | 0.54 | Between |
| TPB.ON | DI\_GC\_EXP | 0.112 | 0.091 | 1.232 | 0.218 | Between |
| TPB.ON | DI\_GC\_WIS | 0.116 | 0.077 | 1.499 | 0.134 | Between |
| TPB.ON | ME\_NP | -0.397 | 0.075 | -5.317 | 0 | Between |
| TPB.ON | MI\_NP | -0.027 | 0.074 | -0.372 | 0.71 | Between |
| TPB.ON | MB\_NP | -0.126 | 0.075 | -1.684 | 0.092 | Between |

```
pander::pander(MIMIC_conf_di_man_results$parameters$stdy.standardized %>% 
                 filter(grepl("ON", paramHeader), grepl("I_EXP", param) | 
                          grepl("I_WIS", param)))
```

| paramHeader | param | est | se | est\_se | pval | BetweenWithin |
| --- | --- | --- | --- | --- | --- | --- |
| TPB.ON | I\_EXP | 0.107 | 0.145 | 0.741 | 0.458 | Between |
| TPB.ON | I\_WIS | 0.457 | 0.148 | 3.084 | 0.002 | Between |

- The standardized confidence intervals of M5 were

```
pander::pander(MIMIC_conf_di_man_results$parameters$ci.stdyx.standardized %>% 
                 filter(grepl("ON", paramHeader), !grepl("I_EXP", param), 
                        !grepl("I_WIS", param)) %>% 
                 select(-low.5, -low5, -up.5, -up5))
```

| paramHeader | param | low2.5 | est | up2.5 | BetweenWithin |
| --- | --- | --- | --- | --- | --- |
| TPW.ON | TR\_PC | -0.277 | -0.216 | -0.155 | Within |
| TPW.ON | KE\_01\_PC | 0.007 | 0.071 | 0.135 | Within |
| TPW.ON | KO\_PC | 0.381 | 0.441 | 0.502 | Within |
| TPB.ON | DI\_GC | -0.276 | -0.066 | 0.145 | Between |
| TPB.ON | DI\_GC\_EXP | -0.066 | 0.112 | 0.291 | Between |
| TPB.ON | DI\_GC\_WIS | -0.036 | 0.116 | 0.267 | Between |
| TPB.ON | ME\_NP | -0.543 | -0.397 | -0.25 | Between |
| TPB.ON | MI\_NP | -0.172 | -0.027 | 0.117 | Between |
| TPB.ON | MB\_NP | -0.274 | -0.126 | 0.021 | Between |

```
pander::pander(MIMIC_conf_di_man_results$parameters$ci.stdy.standardized %>% 
                 filter(grepl("ON", paramHeader), grepl("I_EXP", param) | 
                          grepl("I_WIS", param)) %>% 
                 dplyr::select(-low.5, -low5, -up.5, -up5))
```

| paramHeader | param | low2.5 | est | up2.5 | BetweenWithin |
| --- | --- | --- | --- | --- | --- |
| TPB.ON | I\_EXP | -0.176 | 0.107 | 0.391 | Between |
| TPB.ON | I\_WIS | 0.166 | 0.457 | 0.747 | Between |

- The \(R^2\) were:

```
pander::pander(MIMIC_conf_di_man_results$parameters$r2 %>% 
                 filter(grepl("TPB", param) | grepl("TPW", param)))
```

| param | est | se | est\_se | pval | BetweenWithin |
| --- | --- | --- | --- | --- | --- |
| TPW | 0.375 | 0.029 | 13.044 | 0 | Within |
| TPB | 0.302 | 0.056 | 5.367 | 0 | Between |

### M5a: Confounders of Predictive Effects d-Index without topic knowledge (=topic familiarity) (H3)

```
### MIMIC_conf_di_man Modell  _________________________________
MIMIC_conf_di_man_a <- mplusObject(
  
   TITLE =  "MIMIC_conf_di_man_a",
   
   ANALYSIS =  "TYPE = TWOLEVEL;",
    
   VARIABLE =  "USEVARIABLES = tp_01_np tp_02_np tp_03_np tp_04_np 
                tp_05_np tp_06_np
                tr_pc  ko_pc 
                di_gc di_gc_exp di_gc_wis I_exp I_wis me_np mi_np mb_np;

                WITHIN =  tr_pc  ko_pc;
                BETWEEN = di_gc di_gc_exp di_gc_wis I_exp I_wis me_np mi_np mb_np;
                CLUSTER = IDnum;",
   

   MODEL =      "%WITHIN%
                 TPW BY tp_01_np tp_02_np tp_03_np tp_04_np tp_05_np tp_06_np;

                 TPW ON tr_pc  ko_pc ;

                 %BETWEEN%
                 TPB BY tp_01_np tp_02_np tp_03_np tp_04_np tp_05_np tp_06_np;
                 tp_05_np@0;
                 TPB ON di_gc di_gc_exp di_gc_wis I_exp I_wis me_np mi_np mb_np; ",
   
   OUTPUT = "Standardized CINTERVAL MODINDICES(5);",
   
   rdata = rawdata_long_np)

MIMIC_conf_di_man_a_fit <- mplusModeler(MIMIC_conf_di_man_a, "MIMIC_conf_di_man_a.dat", run = 1)
```

- The fitindices of M5a were: χ² = 130.51, *df* = 69, CFI = 0.973, TLI = 0.965, RMSEA = 0.025, SRMRwithin = 0.024, SRMRbetween = 0.044
- The standardized predictive effects of M5a were:

```
MIMIC_conf_di_man_a_results <- readModels("MIMIC_conf_di_man_a.out")
```

Reading model: MIMIC\_conf\_di\_man\_a.out

```
pander::pander(MIMIC_conf_di_man_a_results$parameters$stdyx.standardized %>% 
                 filter(grepl("ON", paramHeader), !grepl("I_EXP", param), 
                        !grepl("I_WIS", param)))
```

| paramHeader | param | est | se | est\_se | pval | BetweenWithin |
| --- | --- | --- | --- | --- | --- | --- |
| TPW.ON | TR\_PC | -0.222 | 0.03 | -7.306 | 0 | Within |
| TPW.ON | KO\_PC | 0.468 | 0.028 | 16.65 | 0 | Within |
| TPB.ON | DI\_GC | -0.067 | 0.108 | -0.623 | 0.533 | Between |
| TPB.ON | DI\_GC\_EXP | 0.114 | 0.092 | 1.245 | 0.213 | Between |
| TPB.ON | DI\_GC\_WIS | 0.115 | 0.078 | 1.483 | 0.138 | Between |
| TPB.ON | ME\_NP | -0.398 | 0.075 | -5.335 | 0 | Between |
| TPB.ON | MI\_NP | -0.026 | 0.074 | -0.355 | 0.723 | Between |
| TPB.ON | MB\_NP | -0.122 | 0.075 | -1.623 | 0.105 | Between |

```
pander::pander(MIMIC_conf_di_man_a_results$parameters$stdy.standardized %>% 
                 filter(grepl("ON", paramHeader), grepl("I_EXP", param) | 
                          grepl("I_WIS", param)))
```

| paramHeader | param | est | se | est\_se | pval | BetweenWithin |
| --- | --- | --- | --- | --- | --- | --- |
| TPB.ON | I\_EXP | 0.115 | 0.145 | 0.794 | 0.427 | Between |
| TPB.ON | I\_WIS | 0.458 | 0.149 | 3.073 | 0.002 | Between |

- The standardized confidence intervals of M5 were

```
pander::pander(MIMIC_conf_di_man_a_results$parameters$ci.stdyx.standardized %>% 
                 filter(grepl("ON", paramHeader), !grepl("I_EXP", param), 
                        !grepl("I_WIS", param)) %>% 
                 select(-low.5, -low5, -up.5, -up5))
```

| paramHeader | param | low2.5 | est | up2.5 | BetweenWithin |
| --- | --- | --- | --- | --- | --- |
| TPW.ON | TR\_PC | -0.281 | -0.222 | -0.162 | Within |
| TPW.ON | KO\_PC | 0.413 | 0.468 | 0.523 | Within |
| TPB.ON | DI\_GC | -0.279 | -0.067 | 0.145 | Between |
| TPB.ON | DI\_GC\_EXP | -0.065 | 0.114 | 0.293 | Between |
| TPB.ON | DI\_GC\_WIS | -0.037 | 0.115 | 0.267 | Between |
| TPB.ON | ME\_NP | -0.545 | -0.398 | -0.252 | Between |
| TPB.ON | MI\_NP | -0.171 | -0.026 | 0.118 | Between |
| TPB.ON | MB\_NP | -0.27 | -0.122 | 0.025 | Between |

```
pander::pander(MIMIC_conf_di_man_a_results$parameters$ci.stdy.standardized %>% 
                 filter(grepl("ON", paramHeader), grepl("I_EXP", param) | 
                          grepl("I_WIS", param)) %>% 
                 dplyr::select(-low.5, -low5, -up.5, -up5))
```

| paramHeader | param | low2.5 | est | up2.5 | BetweenWithin |
| --- | --- | --- | --- | --- | --- |
| TPB.ON | I\_EXP | -0.169 | 0.115 | 0.4 | Between |
| TPB.ON | I\_WIS | 0.166 | 0.458 | 0.75 | Between |

- The \(R^2\) were:

```
pander::pander(MIMIC_conf_di_man_a_results$parameters$r2 %>% 
                 filter(grepl("TPB", param) | grepl("TPW", param)))
```

| param | est | se | est\_se | pval | BetweenWithin |
| --- | --- | --- | --- | --- | --- |
| TPW | 0.369 | 0.029 | 12.774 | 0 | Within |
| TPB | 0.301 | 0.056 | 5.331 | 0 | Between |

### M6: Confounders of Predictive Effects EBI (H3)

```
### MIMIC_conf_ebi_man Modell  _________________________________

MIMIC_conf_ebi_man <- mplusObject(
  
   TITLE =  "MIMIC_conf_ebi_man",
   
   ANALYSIS =  "TYPE = TWOLEVEL;",
    
   VARIABLE =  "USEVARIABLES = tp_01_np tp_02_np tp_03_np tp_04_np 
                tp_05_np tp_06_np
                tr_pc ke_01_pc ko_pc 
                abs_gc rel_gc abs_gc_exp rel_gc_exp abs_gc_wis rel_gc_wis
                I_exp I_wis me_np mi_np mb_np;

                WITHIN =  tr_pc ke_01_pc ko_pc;
                BETWEEN = abs_gc rel_gc abs_gc_exp rel_gc_exp abs_gc_wis rel_gc_wis
                          I_exp I_wis me_np mi_np mb_np;
                CLUSTER = IDnum;",
   

   MODEL =      "%WITHIN%
                 TPW BY tp_01_np tp_02_np tp_03_np tp_04_np tp_05_np tp_06_np;

                 TPW ON tr_pc ke_01_pc  ko_pc ;

                 %BETWEEN%
                 TPB BY tp_01_np tp_02_np tp_03_np tp_04_np tp_05_np tp_06_np;
                 tp_05_np@0;
                 TPB ON abs_gc rel_gc abs_gc_exp rel_gc_exp abs_gc_wis rel_gc_wis
                        I_exp I_wis me_np mi_np mb_np;",
   
   OUTPUT = "Standardized CINTERVAL MODINDICES(5);",
   
   rdata = rawdata_long_np)

MIMIC_conf_ebi_man_fit <- mplusModeler(MIMIC_conf_ebi_man, "MIMIC_conf_ebi_man.dat", run = 1)
```

- The fitindices of M6 were: χ² = 181.307, *df* = 89, CFI = 0.96, TLI = 0.948, RMSEA = 0.027, SRMRwithin = 0.026, SRMRbetween = 0.047
- The standardized predictive effects of M6 were:

```
MIMIC_conf_ebi_man_results <- readModels("MIMIC_conf_ebi_man.out")
```

Reading model: MIMIC\_conf\_ebi\_man.out

```
pander::pander(MIMIC_conf_ebi_man_results$parameters$stdyx.standardized %>% 
                 filter(grepl("ON", paramHeader), !grepl("I_EXP", param), 
                        !grepl("I_WIS", param)))
```

| paramHeader | param | est | se | est\_se | pval | BetweenWithin |
| --- | --- | --- | --- | --- | --- | --- |
| TPW.ON | TR\_PC | -0.206 | 0.031 | -6.605 | 0 | Within |
| TPW.ON | KE\_01\_PC | 0.073 | 0.033 | 2.221 | 0.026 | Within |
| TPW.ON | KO\_PC | 0.449 | 0.031 | 14.61 | 0 | Within |
| TPB.ON | ABS\_GC | 0.38 | 0.102 | 3.714 | 0 | Between |
| TPB.ON | REL\_GC | 0.153 | 0.092 | 1.667 | 0.096 | Between |
| TPB.ON | ABS\_GC\_EXP | -0.138 | 0.082 | -1.683 | 0.092 | Between |
| TPB.ON | REL\_GC\_EXP | 0.04 | 0.079 | 0.504 | 0.614 | Between |
| TPB.ON | ABS\_GC\_WIS | -0.188 | 0.086 | -2.172 | 0.03 | Between |
| TPB.ON | REL\_GC\_WIS | -0.062 | 0.073 | -0.841 | 0.4 | Between |
| TPB.ON | ME\_NP | -0.403 | 0.069 | -5.829 | 0 | Between |
| TPB.ON | MI\_NP | -0.054 | 0.072 | -0.756 | 0.45 | Between |
| TPB.ON | MB\_NP | -0.092 | 0.073 | -1.265 | 0.206 | Between |

```
pander::pander(MIMIC_conf_ebi_man_results$parameters$stdy.standardized %>% 
                 filter(grepl("ON", paramHeader), grepl("I_EXP", param) | 
                          grepl("I_WIS", param)))
```

| paramHeader | param | est | se | est\_se | pval | BetweenWithin |
| --- | --- | --- | --- | --- | --- | --- |
| TPB.ON | I\_EXP | 0.129 | 0.144 | 0.892 | 0.373 | Between |
| TPB.ON | I\_WIS | 0.434 | 0.151 | 2.884 | 0.004 | Between |

- The standardized confidence intervals of M6 were

```
pander::pander(MIMIC_conf_ebi_man_results$parameters$ci.stdyx.standardized %>% 
                 filter(grepl("ON", paramHeader), !grepl("I_EXP", param), 
                        !grepl("I_WIS", param)) %>% 
                 select(-low.5, -low5, -up.5, -up5))
```

| paramHeader | param | low2.5 | est | up2.5 | BetweenWithin |
| --- | --- | --- | --- | --- | --- |
| TPW.ON | TR\_PC | -0.267 | -0.206 | -0.145 | Within |
| TPW.ON | KE\_01\_PC | 0.009 | 0.073 | 0.137 | Within |
| TPW.ON | KO\_PC | 0.389 | 0.449 | 0.509 | Within |
| TPB.ON | ABS\_GC | 0.179 | 0.38 | 0.58 | Between |
| TPB.ON | REL\_GC | -0.027 | 0.153 | 0.333 | Between |
| TPB.ON | ABS\_GC\_EXP | -0.3 | -0.138 | 0.023 | Between |
| TPB.ON | REL\_GC\_EXP | -0.115 | 0.04 | 0.195 | Between |
| TPB.ON | ABS\_GC\_WIS | -0.357 | -0.188 | -0.018 | Between |
| TPB.ON | REL\_GC\_WIS | -0.205 | -0.062 | 0.082 | Between |
| TPB.ON | ME\_NP | -0.538 | -0.403 | -0.267 | Between |
| TPB.ON | MI\_NP | -0.195 | -0.054 | 0.087 | Between |
| TPB.ON | MB\_NP | -0.235 | -0.092 | 0.051 | Between |

```
pander::pander(MIMIC_conf_ebi_man_results$parameters$ci.stdy.standardized %>% 
                 filter(grepl("ON", paramHeader), grepl("I_EXP", param) | 
                          grepl("I_WIS", param)) %>% 
                 dplyr::select(-low.5, -low5, -up.5, -up5))
```

| paramHeader | param | low2.5 | est | up2.5 | BetweenWithin |
| --- | --- | --- | --- | --- | --- |
| TPB.ON | I\_EXP | -0.154 | 0.129 | 0.411 | Between |
| TPB.ON | I\_WIS | 0.139 | 0.434 | 0.73 | Between |

- The \(R^2\) were:

```
pander::pander(MIMIC_conf_ebi_man_results$parameters$r2 %>% 
                 filter(grepl("TPB", param) | grepl("TPW", param)))
```

| param | est | se | est\_se | pval | BetweenWithin |
| --- | --- | --- | --- | --- | --- |
| TPW | 0.374 | 0.029 | 12.95 | 0 | Within |
| TPB | 0.353 | 0.06 | 5.856 | 0 | Between |

### M6a: Confounders of Predictive Effects EBI, without topic knowledge (=topic familiarity) (H3)

```
### MIMIC_conf_ebi_man Modell  _________________________________

MIMIC_conf_ebi_man_a <- mplusObject(
  
   TITLE =  "MIMIC_conf_ebi_man_a",
   
   ANALYSIS =  "TYPE = TWOLEVEL;",
    
   VARIABLE =  "USEVARIABLES = tp_01_np tp_02_np tp_03_np tp_04_np 
                tp_05_np tp_06_np
                tr_pc ko_pc 
                abs_gc rel_gc abs_gc_exp rel_gc_exp abs_gc_wis rel_gc_wis
                I_exp I_wis me_np mi_np mb_np;

                WITHIN =  tr_pc ko_pc;
                BETWEEN = abs_gc rel_gc abs_gc_exp rel_gc_exp abs_gc_wis rel_gc_wis
                          I_exp I_wis me_np mi_np mb_np;
                CLUSTER = IDnum;",
   

   MODEL =      "%WITHIN%
                 TPW BY tp_01_np tp_02_np tp_03_np tp_04_np tp_05_np tp_06_np;

                 TPW ON tr_pc ko_pc;

                 %BETWEEN%
                 TPB BY tp_01_np tp_02_np tp_03_np tp_04_np tp_05_np tp_06_np;
                 tp_05_np@0;
                 TPB ON abs_gc rel_gc abs_gc_exp rel_gc_exp abs_gc_wis rel_gc_wis
                        I_exp I_wis me_np mi_np mb_np;",
   
   OUTPUT = "Standardized CINTERVAL MODINDICES(5);",
   
   rdata = rawdata_long_np)

MIMIC_conf_ebi_man_a_fit <- mplusModeler(MIMIC_conf_ebi_man_a, "MIMIC_conf_ebi_man_a.dat", run = 1)
```

- The fitindices of M6a were: χ² = 161.163, *df* = 84, CFI = 0.966, TLI = 0.957, RMSEA = 0.026, SRMRwithin = 0.024, SRMRbetween = 0.048
- The standardized predictive effects of M6a were:

```
MIMIC_conf_ebi_man_a_results <- readModels("MIMIC_conf_ebi_man_a.out")
```

Reading model: MIMIC\_conf\_ebi\_man\_a.out

```
pander::pander(MIMIC_conf_ebi_man_a_results$parameters$stdyx.standardized %>% 
                 filter(grepl("ON", paramHeader), !grepl("I_EXP", param), 
                        !grepl("I_WIS", param)))
```

| paramHeader | param | est | se | est\_se | pval | BetweenWithin |
| --- | --- | --- | --- | --- | --- | --- |
| TPW.ON | TR\_PC | -0.209 | 0.031 | -6.849 | 0 | Within |
| TPW.ON | KO\_PC | 0.474 | 0.028 | 17 | 0 | Within |
| TPB.ON | ABS\_GC | 0.38 | 0.102 | 3.714 | 0 | Between |
| TPB.ON | REL\_GC | 0.16 | 0.092 | 1.736 | 0.083 | Between |
| TPB.ON | ABS\_GC\_EXP | -0.14 | 0.082 | -1.701 | 0.089 | Between |
| TPB.ON | REL\_GC\_EXP | 0.037 | 0.079 | 0.473 | 0.636 | Between |
| TPB.ON | ABS\_GC\_WIS | -0.188 | 0.086 | -2.182 | 0.029 | Between |
| TPB.ON | REL\_GC\_WIS | -0.065 | 0.073 | -0.882 | 0.378 | Between |
| TPB.ON | ME\_NP | -0.404 | 0.069 | -5.848 | 0 | Between |
| TPB.ON | MI\_NP | -0.053 | 0.072 | -0.741 | 0.459 | Between |
| TPB.ON | MB\_NP | -0.09 | 0.073 | -1.236 | 0.216 | Between |

```
pander::pander(MIMIC_conf_ebi_man_a_results$parameters$stdy.standardized %>% 
                 filter(grepl("ON", paramHeader), grepl("I_EXP", param) | 
                          grepl("I_WIS", param)))
```

| paramHeader | param | est | se | est\_se | pval | BetweenWithin |
| --- | --- | --- | --- | --- | --- | --- |
| TPB.ON | I\_EXP | 0.136 | 0.144 | 0.939 | 0.348 | Between |
| TPB.ON | I\_WIS | 0.437 | 0.151 | 2.89 | 0.004 | Between |

- The standardized confidence intervals of M6a were

```
pander::pander(MIMIC_conf_ebi_man_a_results$parameters$ci.stdyx.standardized %>% 
                 filter(grepl("ON", paramHeader), !grepl("I_EXP", param), 
                        !grepl("I_WIS", param)) %>% 
                 select(-low.5, -low5, -up.5, -up5))
```

| paramHeader | param | low2.5 | est | up2.5 | BetweenWithin |
| --- | --- | --- | --- | --- | --- |
| TPW.ON | TR\_PC | -0.269 | -0.209 | -0.149 | Within |
| TPW.ON | KO\_PC | 0.419 | 0.474 | 0.528 | Within |
| TPB.ON | ABS\_GC | 0.179 | 0.38 | 0.58 | Between |
| TPB.ON | REL\_GC | -0.021 | 0.16 | 0.34 | Between |
| TPB.ON | ABS\_GC\_EXP | -0.301 | -0.14 | 0.021 | Between |
| TPB.ON | REL\_GC\_EXP | -0.118 | 0.037 | 0.192 | Between |
| TPB.ON | ABS\_GC\_WIS | -0.357 | -0.188 | -0.019 | Between |
| TPB.ON | REL\_GC\_WIS | -0.209 | -0.065 | 0.079 | Between |
| TPB.ON | ME\_NP | -0.54 | -0.404 | -0.269 | Between |
| TPB.ON | MI\_NP | -0.195 | -0.053 | 0.088 | Between |
| TPB.ON | MB\_NP | -0.234 | -0.09 | 0.053 | Between |

```
pander::pander(MIMIC_conf_ebi_man_a_results$parameters$ci.stdy.standardized %>% 
                 filter(grepl("ON", paramHeader), grepl("I_EXP", param) | 
                          grepl("I_WIS", param)) %>% 
                 dplyr::select(-low.5, -low5, -up.5, -up5))
```

| paramHeader | param | low2.5 | est | up2.5 | BetweenWithin |
| --- | --- | --- | --- | --- | --- |
| TPB.ON | I\_EXP | -0.147 | 0.136 | 0.419 | Between |
| TPB.ON | I\_WIS | 0.141 | 0.437 | 0.734 | Between |

- The \(R^2\) were:

```
pander::pander(MIMIC_conf_ebi_man_a_results$parameters$r2 %>% 
                 filter(grepl("TPB", param) | grepl("TPW", param)))
```

| param | est | se | est\_se | pval | BetweenWithin |
| --- | --- | --- | --- | --- | --- |
| TPW | 0.364 | 0.029 | 12.559 | 0 | Within |
| TPB | 0.353 | 0.06 | 5.856 | 0 | Between |
